# Gut Microbiota Participates in Antithyroid Drug Induced Liver Injury Through the Lipopolysaccharide Related Signaling Pathway

**DOI:** 10.3389/fphar.2020.598170

**Published:** 2020-12-17

**Authors:** Jiayu Sun, Fuya Zhao, Baiqiang Lin, Jing Feng, Xin Wu, Yang Liu, Lei Zhao, Biqiang Zhu, Yunwei Wei

**Affiliations:** Department of Oncology and Laparoscopy Surgery, The First Affiliated Hospital of Harbin Medical University, Harbin, China

**Keywords:** gut microbiota, antithyroid drugs, lipopolysaccharide, intestinal barrier, liver injury

## Abstract

**Background:** Drugs can alter the gut microbiota structure, and gut microbiota dysbiosis in turn is correlated with drug side effects through the intestinal endotoxemia hypothesis. Whether antithyroid drugs (including methimazole and propylthiouracil) cause gut microbiota dysbiosis and whether the gut microbiota is correlated with antithyroid drugs induced liver injury is unknown.

**Methods:** Initial Graves’ disease patients were randomly divided into the methimazole group (*n* = 20) and the propylthiouracil group (*n* = 20) and were followed up every 2 weeks; 50 healthy controls were also included. The structure and function of gut microbiota were compared from the cross sectional and longitudinal levels. The correlation between the gut microbiota and clinical parameters was also determined. In addition, Sprague-Dawley rats were randomly allotted into six groups, including four drug groups, which received daily doses of methimazole (1.5 mg/kg/day; 2.5 mg/kg/day) or propylthiouracil (7.5 mg/kg/day; 12.5 mg/kg/day) by oral gavage, and two control groups received the vehicle. In addition to the indexes mentioned above, intestinal barrier-related indexes were also performed.

**Results:** Cross sectional and longitudinal comparison results from both clinical trials and animal studies indicate that antithyroid drugs altered gut microbiota structure; and the liver function related indexes all increased which correlated with gut microbiota. In addition, lipopolysaccharide-related pathways and the lipopolysaccharide concentration in feces and serum all increased after antithyroid drugs administration. These results consistent with the destroyed intestinal barrier in animal study after antithyroid drugs administration.

**Conclusion:** We verified that antithyroid drugs altered gut microbiota structure and that the gut microbiota may in turn be correlated with antithyroid drugs-induced liver injury through the intestinal endotoxemia hypothesis.

## Introduction

Antithyroid drugs (ATDs), including methimazole (MMI) and propylthiouracil (PTU), represent the first-line treatment in Europe, Asia and Latin America (Bartalena et al., 2016; Brito et al., 2016). Liver injury is a side effect of ATDs treatment, although hyperthyroidism itself can lead to different degrees of liver damage (Li et al., 2015). In a study of drug-induced liver injury (DILI) across 308 medical centers in China, MMI, and PTU each accounted for one among 72 death cases (Shen et al., 2019). PTU treatment was even reported to be the third greatest cause of drug-related liver transplantation in the United States (1992–2002) (Russo et al., 2004). However, the specific mechanisms of ATDs-induced liver injury have not yet been fully elucidated, although the cytotoxic substance glyoxal, which is the intermediate product of MMI metabolism, can induce oxidative stress and cell dysfunction (Heidari et al., 2015).

The gut microbiota, a hidden functional organ, has important roles in modulating physiological functions, including immune development and maturation, hematopoiesis and metabolism regulation (Manzo and Bhatt, 2015; Enright et al., 2016; Cani, 2018). Meanwhile, the gut microbiota can be affected by many factors, such as age, diet and drugs. Maier et al. reported that 24% of synthetic drugs with human targets across therapeutic classes inhibited the growth of at least one bacterial strain *in vitro* (Maier et al., 2018). Furthermore, some synthetic drugs antibiotics (Zarrinpar et al., 2018), proton pump inhibitors (PPIs) (Jackson et al., 2016), chemotherapeutics (Montassier et al., 2016), metformin (Wu et al., 2017), and antidepressants (Morgan et al., 2014), altered the composition of the gut microbiota *in vivo* during disease treatment. In turn, the gut microbiota can also metabolize some synthetic drugs. On the one hand, the gut microbiota can modulate the expression of some host cytochrome enzymes, such as CYP8b1 and CYP3a11, which can metabolize drugs (Li et al., 2016). On the other hand, the gut microbiota can directly produce some enzymes that participate in drug absorption, activation and inactivation, which may be closely related to the toxic side effects of drugs (Li and Jia, 2013). The bidirectional drug-microbiome interactions will inevitably contribute to the toxic side effects of many other drugs.

Through intensive studies, the gut microbiota has also been shown to participate in DILI via the following main mechanisms. 1) Drug metabolism: dysbiosis of the gut microbiota induced by a drug in turn leads to drug metabolism into toxic liver metabolites through direct or indirect regulation of the secretion of some enzymes, such as myeloperoxidase and cytochrome P450 (CYP450) (Romano et al., 2015; Aziz et al., 2018). 2) The intestinal endotoxemia hypothesis: a drug may give rise to gut microbiota dysbiosis, intestinal barrier injury and increased intestinal permeability, thus permitting gut microbiota or microbiota-derived product, such as lipopolysaccharide (LPS), translocation into the portal circulation and then transit to the liver. Then intestinal-derived LPS binds to toll-like receptor-4 (TLR-4) on hepatic sinusoidal endothelial cells and Kupffer cells, which can directly or indirectly lead to liver damage; and the gut microbiota transported to the liver can also activate a large number of hepatic inflammatory cells, which synthesize and release many proinflammatory factors in response to membrane antigens or toxic metabolites (such as LPS), causing further liver damage (Chassaing et al., 2014; Gong et al., 2019).

Previous studies have found that gut microbiota in initial Graves’ disease (GD) patients (Init_GD group) differed from that in healthy controls (HCs) though the differential gut microbiota is not exactly the same (Zhou et al., 2014; Ishaq et al., 2018); and some drug could alter the composition of the gut microbiota *in vivo* during disease treatment as mentioned above, ATDs may also cause changes in gut microbiota. Hence, we postulated that the intestinal endotoxemia hypothesis participates in ATDs-induced liver injury.

In clinical treatment, ATDs are often accompanied by adjuvant drugs, such as hepatoprotective drugs and leukogenic drugs, which may also affect the gut microbiota structure. Therefore, to eliminate the effect of hyperthyroidism and adjuvant drugs on the gut microbiota, we designed clinical and animal studies to 1) observe changes in the gut microbiota structure caused by ATDs (including the use or nonuse of ATDs, different ATDs, different ATD doses and different time points) and 2) to preliminarily investigate the roles of the gut microbiota in ATDs-induced liver injury, with the hope of identifying a novel therapeutic strategy to prevent it.

## Materials and Methods

### Ethics Statement, Informed Consented and Clinical Trial Registration

The ethics protocols of the clinical (Eth. 201815) and animal studies (Eth. 2018021) used in this study were all approved by the Ethics Committee of the First Affiliated Hospital of Harbin Medical University in accordance with the relevant regulations and laws. All participants were informed of the nature of the study and provided written informed consent. In addition, a clinical study was registered with the America Clinical Trial Registry (NCT 03433352).

### Recruitment of Init_GD Patients and Healthy Controls

We recruited 40 Init_GD patients from the Endocrine Clinic of the First Affiliated Hospital of Harbin Medical University between December 2017 and December 2018. All Init_GD patients were randomly divided into MMI group (*n* = 20) and PTU group (*n* = 20) and were followed up every two weeks during the first month of ATDs treatment (Treat_GD group). The inclusion criteria were as follows: 18–65 years of age and taking medicine according to prescription. During the same time period, 50 age-, sex- and BMI-matched HCs were recruited from the Health Screening Center (Supplementary Figure S1), and all the participants met the same exclusion criteria (provided in the Supplementary Material).

### Sample Collection and Clinical Parameters

Blood and fecal samples were collected from all Init_GD patients at first diagnosis and 2 and 4 weeks after ATDs administration; as well as HCs. The specific collection and processing methods and a comprehensive description of the clinical parameter analysis are provided in the supplementary information. Blood samples were collected for thyroid function detection (fT3, fT4 and thyroid-stimulating hormone [TSH]) and thyroid autoantibody (Tg-Ab and TPO-Ab), biochemical and serum LPS analyses; fecal samples were used for 16S rRNA gene sequence and fecal LPS detection.

11 pairs of samples failed to be collected, left 159 pairs of samples that were used for further analysis in this study. The samples included 60 pairs from MMI group and 49 pairs from PTU group and 50 pairs of samples from HCs.

### Animals

Forty-five female SPF Sprague-Dawley (SD) rats (6–8 weeks old, weighing 200 ± 10 g) were purchased from the animal experiment center of the Second Affiliated Hospital of Harbin Medical University. The rats were housed in a temperature- and humidity-maintained room (20 ± 3°C, with a relative humidity of approximately 40%) under a 12 h light/dark cycle in stainless steel cages with free access to water and standard rat chow. The rats were allowed to acclimate to their diet and new environment for at least 1 week before initiating the experiment.

### Experimental Setup

Rats were randomly allotted to six groups, including four ATDs groups (*n* = 9 for each group), which received daily doses of 1.5 mg/kg/day MMI (ML group), 2.5 mg/kg/day MMI (MH group), 7.5 mg/kg/day PTU (PL group) or 12.5 mg/kg/day PTU (PH group) by oral gavage. The two control groups (*n* = 4 for Control 1; *n* = 5 for Control 2) received the same doses of vehicle (ordinary drinking water) every day. Control two group rats were introduced later and raised for only 2 weeks to match their body weight with that of the ATDs group because the original control one rats had a heavier body weight, which may affect the gut microbiota structure and intestinal barrier. The study lasted for 11 weeks (the experimental design and timeline naming of the rats are shown in Supplementary Figure S2).

### Sample Collection and Basic Characteristic Measurements

Blood samples were collected for thyroid function detection (fT3, fT4, and TSH) and biochemical and serum LPS analyses. Fecal samples were used for 16S rRNA gene sequence, fecal LPS and fecal calprotectin (CALP) detection. Related tissue samples (including the thyroid, liver, spleen, cecum and total gastrointestinal tract) were also collected. Intestines (including small intestine and colon) electron microscopy analyses, which all correlate with intestinal barrier, were performed (Fang et al., 2018). Thirty-seven fecal samples failed to be collected due to death of some rats; thus, 123 fecal samples were analyzed in this study. The specific sample collection and measurement methods are described in the supplementary information.

### Statistical Analyses

The laboratory examination and analysis of basic animal indicators were performed using the Statistical Package for the Social Sciences (SPSS) version 22.0 (SPSS Inc., 2010 Chicago, IL, United States); mean and corresponding standard error of the mean (SEM) values were calculated, the Wilcoxon rank-sum test was performed as an alternative when data were not normally distributed and Kruskal-Wallis H test was performed when it involved three or more groups. Other specific methods are presented in the supplementary information.

## Results

### Part 1: Clinical Study

#### Study Population and Clinical Parameters

All 90 participants were of Han nationality, born in northeastern China and had a similar dietary structure. The Init_GD, Treat_GD and HCs groups were all age- (*p* = 0.838), sex- (*p* = 0.934) and BMI-matched (*p* = 0.563) (Table 1). With ATDs use, hyperthyroidism of Init_GD patients gradually eased, fT3 and fT4 levels decreased even the TSH level remained low, and Tg-Ab and TPO-Ab levels showed decreasing trends, while the TPO-Ab level showed no significant difference between the Init_GD and Treat_GD groups (*p* = 0.319, Supplementary Table S1). Indicators of liver function, such as ALT, AST and AKP, were higher in both the Init_GD and Treat_GD groups than in the HCs (*p* < 0.0001 among the three indicators), which is consistent with the changes reported in a previous study regarding hyperthyroidism-related liver injury (Li et al., 2015); the Treat_GD group showed more significant differences than the Init_GD group, although ALT and AST levels showed no significant differences between them (*p* = 0.858 and *p* = 0.687). Furthermore, the fecal supernatant LPS, CALP and serum LPS levels showed higher levels in the Init_GD and Treat_GD groups than in the HC group (*p* < 0.0001 among all three indicators). The specific clinical characteristics of GD patients and HCs are shown in Table 1 and Supplementary Table S1.

**TABLE 1:** Clinical and demographic features of GD patients and healthy controls.

Parameters	Init_GD + Treat_GD						HC	p1	p2	p3	p4	p5
	MMI				PTU							
	M0	M2	M4	P0	P2	P4						
Age (year, mean ± SD)	36.1 ± 12.15	36.1 ± 12.15	36.1 ± 12.15	39.3 ± 9.5	37.82 ± 10.97	40.22 ± 9.58	36.58 ± 9.06	0.838	0.925	0.289	1	0.819
SEX (F/M)	17/3	17/3	17/3	16/4	7/4	13/5	40/10	0.934	0.722	0.111	1	0.607
BMI (kg/m^2^, mean ± SD)	21.89 ± 2.94	22.23 ± 2.88	22.59 ± 2.89	22.24 ± 3.09	22.12 ± 2.69	23.02 ± 3.19	21.83 ± 2.13	0.563	0.379	0.539	0.633	0.484
Rash	4/16			3/17			0/50			0.173		
fT3 (pg/mL)	20/0	19/1	13/7	20/0	11/0	18/0	0/50	0	0.026	0.017	0.004	1
fT4 (ng/dl)	20/0	16/4	12/8	20/0	9/2	14/4	0/50	0	0	0.385	0.004	0.05
TSH (uIU/mL)	20/0	20/0	19/1	20/0	11/0	18/0	0/50	0	1	1	1	1
Tg-Ab (IU/ml)	18/2	12/8	13/7	18/2	6/5	14/4	0/50	0	0.004	0.578	0.087	0.09
TPO-Ab (IU/ml)	18/2	18/2	18/2	17/3	10/1	15/3	0/50	0	1	0.712	1	1
WBC (109/L)	2/18	2/18	3/17	0/20	0/11	1/17	0/50	0.146	1	0.389	1	0.592
NEUT (109/L)	2/18	2/18	3/17	1/19	0/11	2/16	0/50	0.048	0.743	0.69	1	0.599
LYMPH(109/L)	0/20	0/20	1/19	0/20	0/11	0/18	0/50	1	1	1	1	1
MONO(109/L)	0/20	0/20	0/20	0/20	0/11	0/18	0/50	1	1	1	1	1
EO (109/L)	0/20	0/20	0/20	0/20	0/11	0/18	0/50	1	1	1	1	1
BASO(109/L)	0/20	0/20	0/20	0/20	0/11	0/18	0/50	1	1	1	1	1
RBC(1012/)	2/18	5/15	4/16	5/15	3/8	6/12	0/50	0	0.352	0.579	0.589	0.919
HCT (%)	0/20	0/20	0/20	0/20	1/10	0/18	0/50	1	1	1	1	0.224
MCV(fL)	11/9	10/10	11/9	8/12	0/11	8/10	0/50	0	0.689	0.05	1	0.02
MCH(pg)	7/13	7/13	9/11	3/17	0/11	2/16	0/50	0	1	0.002	0,842	0.521
MCHC(g/L)	4/16	3/17	4/16	0/20	0/11	1/17	0/50	0.025	1	0.126	1	0.592
PCT (%)	0/20	0/20	0/20	0/20	0/11	0/18	0/50	1	1	1	1	1
MPV(fL)	0/20	0/20	0/20	0/20	0/11	0/18	0/50	1	1	1	1	1
HGB (g/L)	1/19	1/19	0/20	1/19	3/8	2/16	0/50	0.075	0.265	0.076	1	0.163
PLT (109/L)	4/16	7/13	6/14	3/17	3/8	2/16	0/50	0	0.352	0.154	0.675	0.544
ALT (U/L)	8/12	10/10	7/13	3/17	2/9	2/16	0/50	0	0.829	0.011	0.72	0.881
AST (U/L)	1/19	6/14	4/16	1/19	1/10	0/18	0/50	0.003	0.127	0.019	0.15	0.694
GGT (U/L)	3/17	1/19	5/15	7/13	4/7	4/14	0/50	0	0.634	0.199	0.265	0.623
TP (g/L)	1/19	2/18	2/18	1/19	0/11	1/17	0/50	0.146	1	0.389	1	1
ALB (g/L)	0/20	0/20	0/20	0/20	0/11	0/18	0/50	1	1	1	1	1
GLB (g/L)	1/19	4/16	5/15	5/15	4/7	3/15	0/50	0	0.335	0.874	0.305	0.528
AKP(U/L)	7/13	8/12	11/9	12/8	7/4	14/4	0/50	0	0.323	0.038	0,515	0.466
TBIL (umol/L)	2/18	0/20	0/20	5/15	3/8	5/13	0/50	0.005	0.401	0.001	0.322	1
DBIL (umol/L)	7/13	2/18	5/15	8/12	4/7	7/11	0/50	0	0.28	0.056	0.207	1
IBIL (umol/L)	2/18	0/20	1/19	5/15	3/8	5/13	0/50	0.004	0.58	0.003	0.766	1
TBA (umol/L)	1/19	1/19	0/20	0/20	0/11	0/18	0/50	0.725	1	1	1	1
PA (mg/L)	10/10	4/16	4/16	7/13	1/10	2/16	0/50	0	0.003	0.336	0.087	0.161

The measurement data and numeration data were statistically analyzed with t test (or one-way ANOVA for multi group comparison) and χ^2^ test, respectively. Measurement data are expressed as the mean ± SD and numeration data are expressed as rate or composition (n1/n2 means abnormal/normal). F/M, female/male; BMI, body mass index; fT3, free triiodothyronine; fT4, free thyroxine; TSH, thyroid-stimulating hormone; Tg-Ab, antithyroglobulin antibody; TPO-Ab, antithyroperoxidase antibody; WBC, white blood cell; NEUT, neutrophils; LYMPH, lymphocytes; MONO, monocytes; EO, eosinophils; BASO, basophils; RBC, red blood cell; HCT, hematocrit; MCV, mean corpuscular volume; MCH, mean corpuscular hemoglobin; MCHC, mean corpuscular hemoglobin concentration; PCT, platelet crit; MPV, mean platelet volume; HGB, hemoglobin; PLT, platelet; ALT, alanine aminotransferase; AST, aspartate transaminase; GGT, gamma-glutamyl transpeptidase; TP, total protein; ALB, albumin; GLB, globulin; AKP, alkaline phosphatase; TBIL, total bilirubin; DBIL, direct bilirubin; IBIL, indirect bilirubin; TBA, total bile acid; PA, pre-albumin and SD, standard deviation. Init_GD: including M0 and P0; Treat_GD: including MMI and PTU; p1: *p* Value of Init_GD vs. Treat_GD vs. HC; p2: *p* Value of Init_GD vs. Treat_GD; p3: *p* Value of MMI vs. PTU; p4: *p* Value of M0 vs. M2 vs. M4; p5: *p* Value of P0 vs. P2 vs. P4.

### Gut Microbial Structure Analysis

#### Sequencing Characteristics

From the 159 samples, 12, 346, 125 high-quality sequences were selected based on barcode- and primer-sequence filtering, with an average of 77,649 (ranging from 50,651 to 92,227) sequences per barcoded sample. A total of 3,442 OTUs were delineated at a 97% similarity level. The rarefaction curve of all samples reached a plateau (Supplementary Figure S3A), and Good’s coverage for all samples was greater than 99.3%, indicating a satisfactory gut microbiota sequencing depth. Furthermore, we divided these samples into 11 subgroups according to the aims of our research; the specific information is shown in Supplementary Table S2. The aims were as follows:

## Part 1: To Investigate Whether Antithyroid Drugs Could Cause Changes in the Gut Microbiota Structure (Treat_GD vs. Init_GD vs. Healthy Control; Treat_GD vs. Init_GD; Treat_GD vs. Healthy Control; Init_GD vs. Healthy Control)

### Alpha and Beta Diversity

**FIGURE 1:**
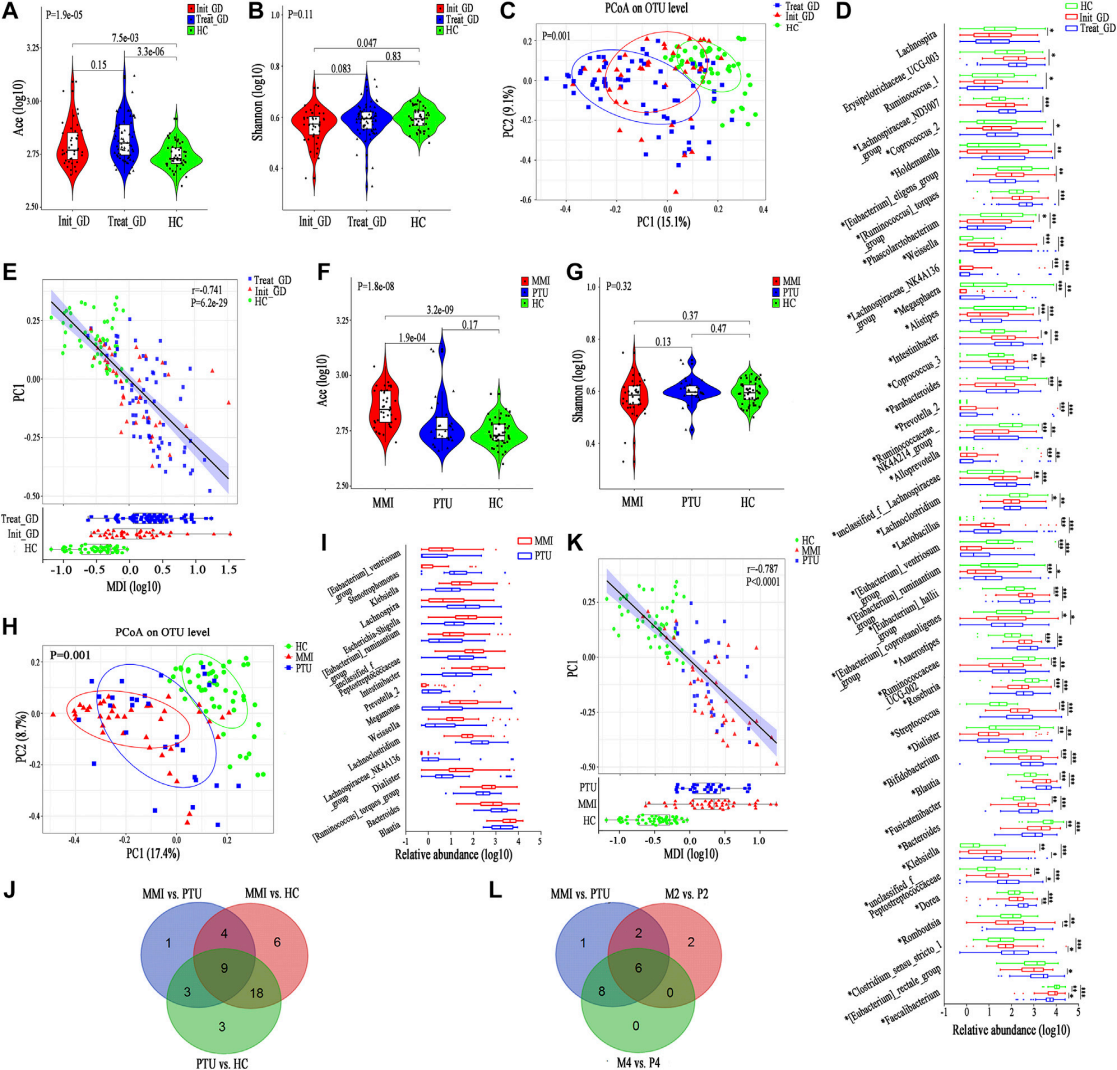
Shift in the gut microbiota diversity and structure after ATDs administration. **(A,B)** Violin plot of the alpha diversity indexes for richness (Ace index) and evenness (Shannon index) of the gut microbiota among the Init_GD, Treat_GD, and HC groups. **(C)** PCoA based on the Bray-Curtis distance showing different clusters among the three groups (*p* = 0.001). **(D)** Comparisons of the relative abundances among differentially abundant genera by Kruskal-Wallis H test (*p* < 0.05). **(E)** The MDI in the three groups and spearman correlation between the MDI and PC1 of the PCoA. The MDI is defined as the log of [total abundance among genera increased in Init_GD or Treat_GD] divided by [total abundance among genera decreased in Init_GD or Treat_GD]. **(F,G)** Violin plot of the alpha diversity Ace index and Shannon index of the gut microbiota among the MMI, PTU and HC groups. **(H)** PCoA based on the Bray-Curtis distance showing different clusters among the MMI, PTU, and HC groups (*p* = 0.001). **(I)** Comparisons of the relative abundances among differentially abundant genera by Mann-Whitney U-tests (*p* < 0.05). **(J)** Venn diagram demonstrating the number of shared and specific differentially abundant genera of every pair of groups among the MMI, PTU, and HC groups. **(K)** The MDI in the MMI, PTU, and HC groups and spearman correlation between the MDI and PC1 of the PCoA. **(L)** Venn diagram demonstrating the number of shared and specific differentially abundant genera in the holistic comparison and corresponding specific time points between MMI and PTU. *p indicates a *p* value < 0.05 among the three groups, p* indicates a *p* value < 0.05 between each pair of groups. *p value < 0.05; **p value < 0.01; ***p value < 0.001.

First, we compared the gut microbiota alpha diversity of the three groups (Init_GD, Treat_GD and HC). The Ace index increased in the Init_GD and Treat_GD groups compared with that in the HC group, and the Treat_GD group showed more higher (Init_GD vs. HC, *p* = 7.5e-03; Treat_GD vs. HC, *p* = 3.3e-06, Figure 1A). Similarly, the Shannon index was also higher in Treat_GD group than in the Init_GD group though there was no significant difference between them. However, both the two groups was lower in the Init_GD and Treat_GD groups than in the HC group (Init_GD vs. HC, *p* = 0.047; Treat_GD vs. HC, *p* = 0.83, Figure 1B). In addition, the other alpha diversity indexes are shown in Supplementary Table S2.

To evaluate the similarities and differences among microbiota communities, PCoA was performed, which illustrated that the Init_GD and Treat_GD groups both deviated from the HC group. The Treat_GD group showed a greater deviation from the HC group than the Init_GD group (*p* = 0.001, Figure 1C), and the Treat_GD group partially deviated from the Init_GD group when compared separately (*p* = 0.064, Supplementary Figure S3B).

The alpha and beta diversity results all indicated that the gut microbiota structures of the Init_GD and Treat_GD groups differed from that of the HC group and that the gut microbiota structure of the Treat_GD group differed from that of the Init_GD group (i.e., the gut microbiota varied after ATDs treatment), although the difference was not as obvious as those between the GD groups and the HC group.

### Differentially Abundant Species Analysis

To identify the specific communities associated with ATDs treatment, we further compared the gut microbiota structure between the Treat_GD and Init_GD groups; 11 species, representing four families (*p* < 0.05, Supplementary Figure S3C) and 7 genera (*p* < 0.05, Figure 1D), were significantly differentiated. *Faecalibacterium* and *Clostridium_sensu_stricto_1* showed lower abundance in the Treat_GD group than in the Init_GD group, while *Eubacterium_rectale*, *Romboutsia* and *Dorea* showed higher abundance in the Treat_GD group at the genus level. Then, we compared the gut microbiota structure of the Init_GD and Treat_GD groups with that of the HC group, and 40 differentially abundant genera were revealed in the Treat_GD group vs. the HC group. Thirty-one differentially abundant genera were revealed in the Init_GD group vs. the HC group, and 30 of them were also differentially abundant between the Treat_GD group and the HC group (Supplementary Figure S3D). Most of the 30 differentially abundant species showed an increasing or decreasing trend after ATDs treatment based on the change in the Init_GD group compared with the HC group, which may be because the previous the gut microbiota structure changes were aggravated by medication (Figure 1D). For example, *Bacteroides* abundance was significantly decreased in the Init_GD and Treat_GD groups compared with that in the HC group, and that in the Treat_GD group decreased more, though the difference showed no statistical significance between the Treat_GD and Init_GD groups. Similarly, *Blautia* abundance showed the opposite trend, which illustrated that the gut microbiota structure in the Init_GD group differed from that in the HC group and that *Blautia* abundance in the Treat_GD group showed more deterioration (Figure 1D).

In the present study, 44 differentially abundant species, representing two phyla, 11 families (*p* < 0.05, Supplementary Figure S3C) and 31 genera (*p* < 0.05, Figure 1D), were revealed between the Init_GD group and the HC group. However, the differentially abundant species were not exactly the same among different studies. For example, at the phylum level, our present and previous study both showed that the *Actinobacteria* and *Firmicutes* proportions were increased while the *Bacteroidetes* proportion was decreased in Init_GD patients compared with those in HCs (Supplementary Figure S3C). In addition, an animal study on Graves’ ophthalmopathy (GO) also showed higher *Firmicutes* and lower *Bacteroidetes* abundance in the GO group than in the control group (Masetti et al., 2018), while two other clinical studies on hyperthyroidism and GO showed higher *Bacteroidetes* and lower *Firmicutes* abundances (Ishaq et al., 2018; Shi et al., 2019).

The microbial dysbiosis index (MDI, the MDI is defined as the log of [total abundance in genera increased in disease group] over [total abundance of genera decreased in disease group]) and the *Firmicutes*/*Bacteroidetes* ratio (F/B ratio) both indicate gut microbiota disturbance, and the F/B ratio is even considered to be representative of health status (Turnbaugh et al., 2006; Gevers et al., 2014). Similar to the change in the alpha diversity Ace index, both of these indexes were significantly higher in the Init_GD and Treat_GD groups than in the HC group, and these indexes were more higher in the Treat_GD group and significantly correlated with PC1 of the PCoA (MDI, *r* = −0.741, *p* = 6.2e-29; F/B ratio, *r* = −0.624, *p* = 1.6e-18) (Figure 1E; Supplementary Figure S3E). These results showed that dysbiosis occurred in the Init_GD and Treat_GD groups, and the Treat_GD group showed greater disturbances than the Init_GD group.

## Part 2: To Investigate Whether Different Antithyroid Drugs Cause Different Gut Microbiota Structure Changes (Methimazole (Including M2 and M4) vs. Propylthiouracil (Including P2 and P4) vs. Healthy Control; Methimazole vs. Propylthiouracil; Methimazole vs. Healthy Control; Propylthiouracil vs. Healthy Control)

It is well known that the pharmacodynamics and toxicology differ between the MMI and PTU, and whether these differences are correlated with the gut microbiota is unknown (Franklyn, 2009). Therefore, we first performed a holistic analysis of the gut microbiota between the MMI, PTU and HC groups and then performed detailed comparisons at each specific time point.

### Alpha and Beta Diversity

The gut microbiota Ace index was significantly increased in the MMI group compared with that in the PTU group (*p* = 1.9e-04), and both the MMI and PTU groups showed higher Ace indexes than the HC group (MMI vs. HC, *p* = 3.2e-09; PTU vs. HC, *p* = 0.17; Figure 1F). The Shannon index was not significantly different among the three groups (*p* = 0.32). Interestingly, the MMI group showed a lower Shannon index, while the PTU group showed a higher Shannon index than the HC group (MMI vs. HC, *p* = 0.37; PTU vs. HCs *p* = 0.47; Figure 1G). The other alpha diversity indexes are shown in Supplementary Table S2. PCoA also revealed differences in the distributions of the three groups (*p* = 0.001): the PTU group was more similar to the HC group than the MMI group (Figure 1H), and the MMI group significantly deviated from the PTU group when they were compared separately (*p* = 0.026, Supplementary Figure S4A).

### Differentially Abundant Species Analysis

Both alpha and beta diversity results showed that the gut microbiota structure in the MMI group differed from that in the PTU group. We then compared the gut microbiota composition of the MMI and PTU groups, and 25 discriminative species, representing two phyla, six families (*p* < 0.05, Supplementary Figure S4B), and 17 genera (*p* < 0.05, Figure 1I), were revealed. At the phylum level, a higher proportion of *Firmicutes* was shown in the MMI group than in the PTU group, while *Bacteroidetes* was more prevalent in the PTU group. The corresponding family, Enterobacteriaceae*,* was more prevalent in the MMI group, whereas *Bacteroidaceae* was more prevalent in the PTU group. At the genus level, *Blautia* and *Escherichia-Shigella* were enriched in the MMI group, while *Bacteroides*, Lachnospiraceae*_NK4A136_group* and *Lachnoclostridium* were enriched in the PTU group. To further illustrate the different effects of different ATDs on the gut microbiota structure, we compared the gut microbiota of the MMI and PTU groups with that of the HC group, and the differentially abundant species among the groups are shown in a Venn diagram. An additional six differentially abundant genera were unique in MMI vs. those in HC, and three genera were unique in PTU vs. those in HC, which further reflects the difference in the microbiota between the MMI and PTU groups (Figure 1J; Supplementary Table S3). Similarly, the MDI and F/B ratio changes were also consistent with the gut microbiota changes. Both the MDI and F/B ratio were higher in the MMI and PTU groups than in the HC group, and the MMI group exhibited a higher MDI and F/B ratio than the PTU group. Additionally, the two indexes correlated with PC1 (MDI, *r* = −0.787, *p* < 0.0001, Figure 1K; F/B ratio, *r* = −0.651, *p* = 1.15e-15, Supplementary Figure S4C), which showed that the gut microbiota structure was destroyed in both the MMI and PTU groups and that the MMI group showed greater dysbiosis than the PTU group.

## Part 3: To Investigate the Longitudinal Changes in the Gut Microbiota Structure Caused by Antithyroid Drugs at Different Time Points

As shown above, the ATDs and different ATDs all contributed to an altered gut microbiota structure, and our previous study showed that the gut microbiota partially recovered in euthyroid GD patients compared with that in Init_GD patients. However, the short-term longitudinal changes in the gut microbiota structure during drug treatment (whether they become more disordered and then normalize or they gradually normalize) are unknown. We compared the gut microbiota changes in the MMI group and the PTU group over the time course of treatments.

## Longitudinal Changes in the Gut Microbiota Structure During Methimazole Treatment (M0 vs. M2 vs. M4 vs. Healthy Control; M0 vs. M2 vs. M4 (including Separate Comparisons of Each Pair of Groups))

### Alpha and Beta Diversity

**FIGURE 2:**
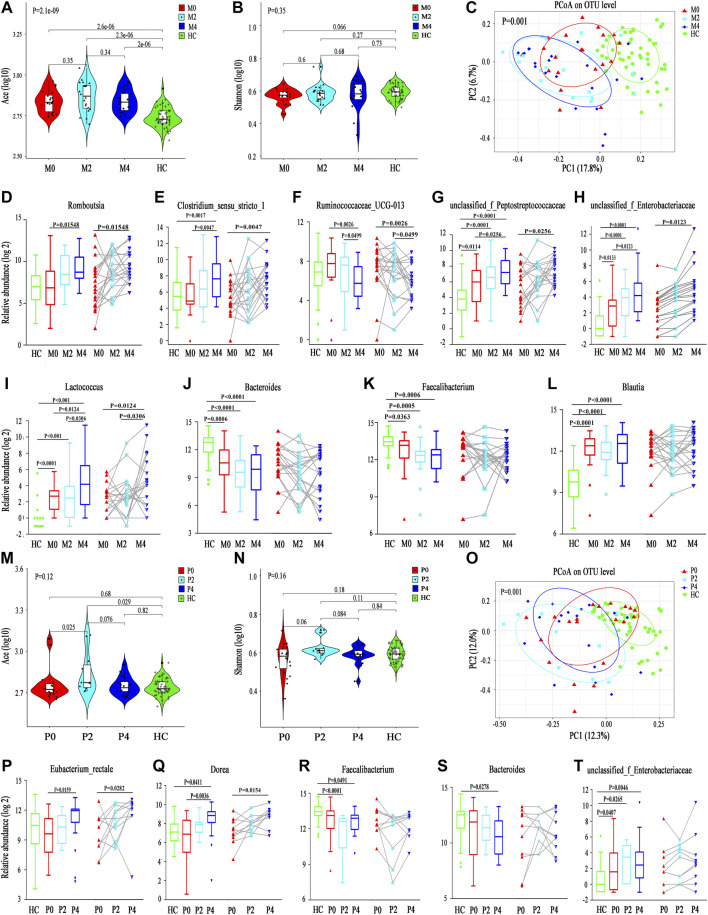
Longitudinal changes in the gut microbiota diversity and structure between MMI and PTU. **(A,B)** Violin plot of the alpha diversity indexes for richness (Ace index) and evenness (Shannon index) of the gut microbiota among the M0, M2, M4, and HC groups. **(C)** PCoA analysis based on the Bray-Curtis distance showing different clusters among the four groups (*p* = 0.001). **(D–L)** Unpaired test (left) and paired test (right) comparisons of the relative abundances in each pair of the M0, M2, M4, and HC groups. **(M,N)** Violin plot of the alpha diversity Ace index and Shannon index of the gut microbiota among the P0, P2, P4, and HC groups. **(O)** PCoA based on the Bray-Curtis distance showing different clusters among the four groups (*p* = 0.001). **(P-T)** Unpaired t test (left) and paired t test (right) comparisons of the relative abundances in each pair of the P0, P2, P4, and HC groups.

The Ace index was significantly higher in the M0, M2 and M4 groups than in the HC group (M0 vs. HC, *p* = 2.6e-6; M2 vs. HC, *p* = 2.3e-6; M4 vs. HC, p = 2e-6), although no significant differences were found among the M0, M2 and M4 groups. The Ace index increased at the 2nd week after taking MMI, but interestingly, it declined at the 4th week, which may be because of the partial recovery of thyroid function (Figure 2A). The Shannon index also showed a similar trend among the M0, M2 and M4 groups, although no significant differences were found among groups or in comparison with the HC group (*p* = 0.35). Nonetheless, the M0 group showed a lower Shannon index than the HC group, which is consistent with a lower alpha diversity in Init_GD patients than in HCs (Figure 2B; Supplementary Table S2). Consistent with the alpha diversity results, PCoA showed that the M0, M2, and M4 groups clearly deviated from the HC group (*p* = 0.001); the M2 and M4 groups were farther from the HC group than the M0 group; while the M2 and M4 groups were more similar to one another (Figure 2C). The other alpha diversity indexes are shown in Supplementary Table S2.

### Differentially Abundant Species Analysis

Differentially abundant species analysis was also performed to explore differences among different time points (M0-M2-M4), which included comparisons between each pair of time points. Only six discriminative features (representing one family and five genera) (Supplementary Figure S4D) with a prevalence >10% and a maximum proportion >0.2% were found. However, as we mentioned above, PCoA showed that the M0, M2, and M4 groups deviated from one another, which may be because of the low abundance of differentially abundant species playing a dominant role. In truth, an additional 20 discriminative features, representing four families and 16 genera, with a prevalence >10% (low-abundance microbiota constituents), are also shown in Supplementary Figures S4E, F. Among them, the abundance of microbiota constituents *Romboutsia* and *Clostridium_sensu_stricto_1* increased, while *Ruminococcaceae_UCG-013* abundance decreased, according to a comparison among the three time points (Figures 2D–I). Some genera, such as *Faecalibacterium* and *Blautia*, had no statistical significance in comparisons among the three time points (M0 vs. M2 vs. M4) but were significantly different from those in HCs and showed longitudinal changes (Figures 2J–L).

## Longitudinal Changes in the Gut Microbiota Structure During Propylthiouracil Treatment (P0 vs. P2 vs. P4 vs. Healthy Control; P0 vs. P2 vs. P4, Including Separate Comparisons of Each Pair of Groups)

### Alpha and Beta Diversity

The alpha diversity longitudinal change trend in the PTU group was similar to that in the MMI group among the P0, P2 and P4 time points (increased at the 2nd week and decreased at the 4th week); however, the Ace and Shannon indexes showed no significant differences among the P0, P2 and P4 time points compared with those of HCs except for an increased Ace index at P2 (Ace: P2 vs. HC, *p* = 0.029; P2 vs. P0, *p* = 0.025; Figures 2M,N). Consistent with the reduced alpha diversity in Init_GD patients, the two indexes were lower at P0 than in HCs. PCoA also showed that P0, P2, and P4 clearly deviated from HCs (*p* = 0.001, Figure 2O). P2 and P4 were farther from HCs than P0 and P2 was the furthest from HCs.

### Differentially Abundant Species Analysis

Differentially abundant species analysis among the three time points (P0-P2-P4) showed only two discriminative species (*Eubacterium_rectale* and *Dorea*) with a prevalence >10% and a maximum proportion >0.2%, and both species showed increasing trends over the time of PTU use (Figures 2P,Q; Supplementary Figure S4G). However, an additional 28 discriminative features, representing one phylum, 10 families and 17 genera, were observed among low-abundance microbiota constituents (Supplementary Figures S4H–J). Similar to in the MMI group, *Faecalibacterium*, *Bacteroides* and *unclassified_f_*Enterobacteriaceae, had no statistical significance in comparisons among the three time points (P0 vs. P2 vs. P4) but were significantly different from HCs and showed longitudinal changes are shown in Figures 2R–T; and the low-abundance discriminative microbiota explained separation result of the PCoA.

In summary, the longitudinal changes in the gut microbiota structure in the MMI and PTU groups reflect not only the effect of ATDs on the microbiota but also the effects of different ATDs; for example, *Romboutsia* abundance increased mainly in the MMI group, while *Eubacterium_rectale* and *Dorea* abundances increased mainly in the PTU group. In addition, microbiota dysbiosis developed in both the Init_GD and Treat_GD groups, and the dysbiosis was aggravated at the 2nd week and showed partial recovery by the 4th week.

### Associations Between the Gut Microbiota and Clinical Indexes

**FIGURE 3:**
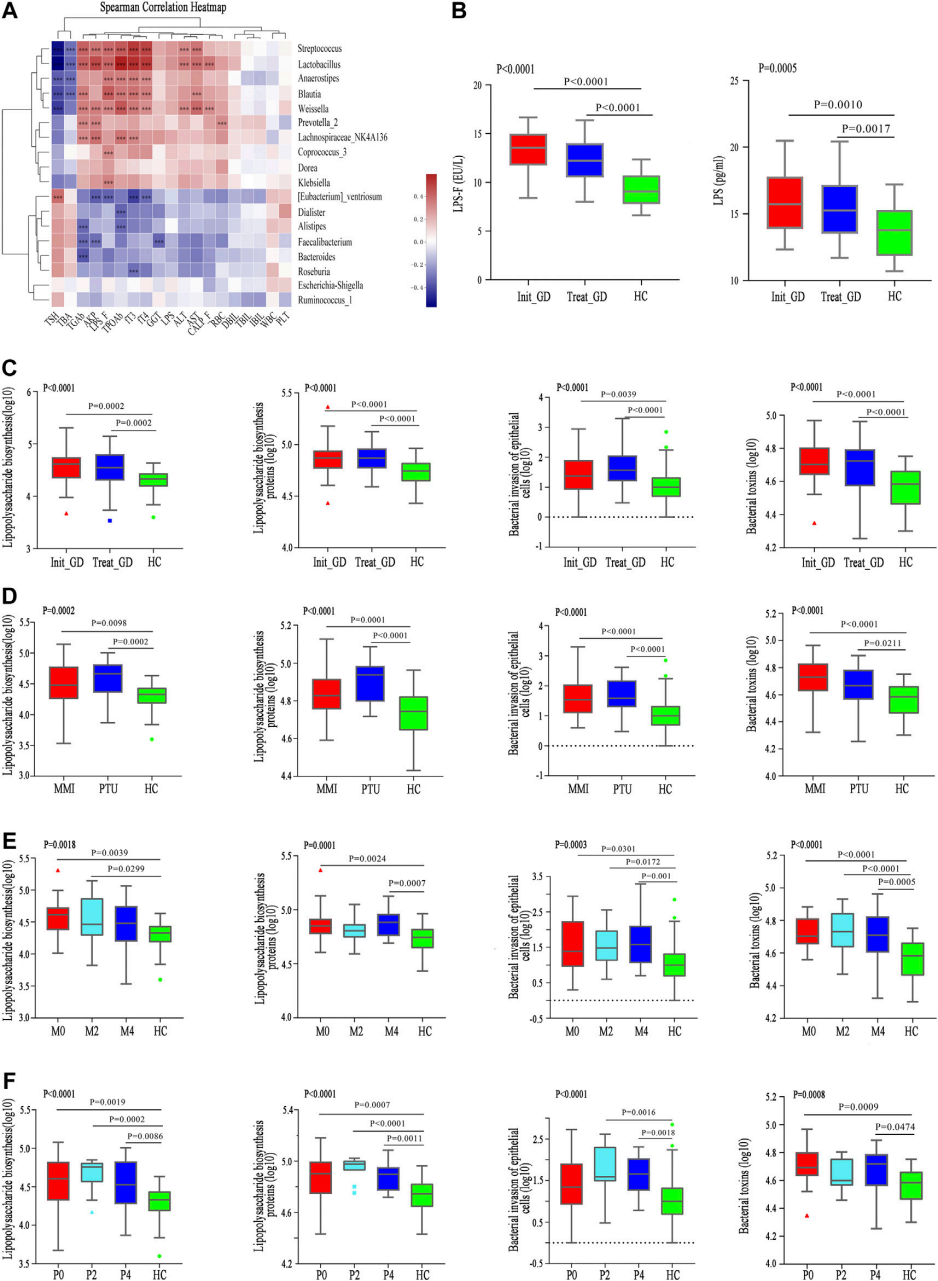
Correlation between differential microbiota and basal indicators, and change of KEGG and gut barrier related indexes. **(A)** Spearman correlation analysis of 19 clinical indicators and 18 differential genera; color intensity represents the magnitude of correlation. Red, positive correlations; blue, negative correlations. *p value < 0.05; **p value < 0.01; ***p value < 0.001. **(B)** LPS concentration in serum (LPS) and fecal supernatant (LPS_F). **(C–F)** The change of pathway Lipopolysaccharide biosynthesis, Lipopolysaccharide biosynthesis proteins, Bacterial invasion of epithelial cells, and Bacterial toxins among groups with ATDs, different ATDs and different time points.

To explore the effect of changes in the gut microbiota structure on phenotypes, we further investigated the correlation between the relative abundance of all differentially abundant genera (*n* = 47) and clinical parameters (*n* = 38) using Spearman’s correlation analysis (Supplementary Figure S5). The abundance levels of Treat_GD group-enriched genera, including *Blautia*, *Streptococcus* and *Dorea,* were positively correlated with some liver function-related indicators, such as ALT, AST and AKP; however, these indicators were negatively correlated with some HC-enriched genera, including *Faecalibacterium* and *Bacteroides* (Figure 3A). These microbiota constituents all participate in liver function regulation, which may also be correlated with ATDs-induced liver injury (Chen et al., 2014; Shen et al., 2017; Meng et al., 2018). In addition, the clinical parameters fT3, fT4, Tg-Ab, and TPO-Ab were positively correlated with *Lactobacillus*, *Streptococcus*, *Blautia,* etc., and negatively correlated with *Roseburia*, *Dialister*, *Alistipes,* etc., while TSH showed opposite results. Moreover, the changes in LPS-producing microbiota constituents, such as *Klebsiella* and *Escherichia-Shigella*, were positively correlated with the LPS variations in the fecal supernatant and serum (Figure 3A)*. Klebsiella* abundance was also positively correlated with liver function indexes, such as ALT, AST and AKP.

### Functional Alterations in the Gut Microbiota

As mentioned above, both the gut microbiota and clinical parameters were altered after ATDs treatment and showed a certain correlation. The role that the gut microbiota plays is unknown, and thus, Phylogenetic Investigation of Communities by Reconstruction of Unobserved States (PICRUSt) of all gut microbiota, which can predict the function of gut microbiota, was performed. We hypothesized that the disrupted gut microbiota plays key roles in ATDs-induced liver injury through the intestinal endotoxemia hypothesis, and the mainly related four metabolic pathways were analyzed.

Lipopolysaccharide biosynthesis (*p* < 0.0001) and Lipopolysaccharide biosynthesis protein (*p* < 0.0001) pathways were more enriched in the Init_GD and Treat_GD groups than in the HC group, which consistent with LPS variations in the fecal supernatant and serum (Figures 3B,C). In addition, Bacterial invasion of epithelial cells and Bacterial toxins pathways, which are associated with bacterial translocation from the intestine, impaired metabolic homeostasis and, subsequently, chronic low-grade inflammation, were also evaluated and were more enriched in the Init_GD and Treat_GD groups than in the HC group (Figure 3C). Similarly, PICRUSt was performed among different ATDs and different time points. All four pathways showed higher activity in the MMI and PTU groups than in the HC group, although there were no significant differences among them (Figure 3D). The four pathways showed higher activation in the Init_GD group, and they exhibited generally increasing trends with MMI or PTU use (Figures 3E,F).

In summary, consistent with the change in the gut microbiota structure, the gut microbiota function may changed along with the change of gut microbiota structure, and these changes were correlated with clinically relevant phenotypes. In particular, the increased activity of pathways associated with the intestinal endotoxemia hypothesis is consistent with the change in LPS concentration in the fecal supernatant and serum, which serves as a preliminary clinical validation of our hypothesis that the gut microbiota participates in ATDs-induced side effects through the intestinal endotoxemia hypothesis.

### Part 2: Animal Study

The animal studies not only repeated the above clinical study but also provided another intervention (i.e., different doses) and displayed related organizational structural changes.

#### Study Subjects and Baseline Indicators

Female age- and weight-matched (6–8 weeks old, weighing 200 ± 10 g) SD rats were purchased from the animal experiment center of the Second Affiliated Hospital of Harbin Medical University and raised in the same environment. Thyroid function decreased gradually with the use of ATDs. At the 4th week, fT3 and fT4 levels showed no significant differences when compared with baseline, while the TSH level showed differences among subgroups, such as between the MH and PL groups. At the 6th week, fT3 and fT4 levels also showed differences from the baseline in some subgroups, indicating the occurrence of hypothyroidism, and fT3, fT4, and TSH levels all showed significant differences from the baseline at the 10th week in all groups (Table 2). Consistent with the serum results, the rats in the ATDs group exhibited rough hair, reduced food intake and relatively slow movement with the development of hypothyroidism. The rat body weight in the Control one group increased, while the rat body weight in the ATDs group decreased, and the PTU group decreased more (Table 2). However, it is worth noting that body weight is usually increased in rats with hypothyroidism (Kyriacou et al., 2019); this conflicting result may be because of the reduced food intake and malnutrition after ATDs administration, which is consistent with the lower ALB level in the ATDs group than in the control group (Supplementary Table S4). The changes in the liver, spleen, thyroid and gastrointestinal weight are shown in Supplementary Figure S6A. Interestingly, after group matching and adjusting for weight, we found that the gastrointestinal tissue weight was significantly correlated with body weight. In contrast, the thyroid weight was higher in the ATDs group than in the control group, while the liver and spleen weights were lower in the ATDs group. In the high-dose group, both MMI- and PTU-administered rats showed obvious trends though the liver weight was greater in the high-dose group than in the low-dose group. The increase in thyroid weight may be due to reactive hyperplasia, which is consistent with the enlarged volume of the thyroid at the 2nd week compared with that in the control group (Supplementary Figure S6B) (Shibutani et al., 2009). The decrease in liver and spleen weight may be due to ATDs-induced injury, which is consistent with the results for the following biochemical indicators (Cano-Europa et al., 2011; Fukui et al., 2013).

**TABLE 2:** Basic parameters of SD rats.

	Group	Baseline	4th week	6th week	10th week
fT3	ML	6.09 ± 0.75	6.39 ± 0.54	5.61 ± 0.85	3.8 ± 0.59***
	MH	5.76 ± 0.59	5.66 ± 0.46	4.56 ± 0.32**	3.82 ± 0.62***
	PL	6.84 ± 0.63	6.14 ± 0.71	5.97 ± 0.63*	4.88 ± 0.49***
	PH	5.42 ± 0.45	6 ± 0.87	5.21 ± 0.48	4.43 ± 0.7**
	ATDs	6.06 ± 0.8	6.05 ± 0.69	5.43 ± 0.78**	4.35 ± 0.73***
fT4	ML	19.9 ± 2.03	19.47 ± 2.69	17.68 ± 1.67*	14.87 ± 2.49**
	MH	20.97 ± 3.06	18.53 ± 1.81	13.49 ± 2.32**	13.61 ± 1.34***
	PL	21.42 ± 2.06	19.76 ± 2.26	19.7 ± 2.86	16.15 ± 2.37***
	PH	19.73 ± 3.03	20.81 ± 2.23	17.14 ± 2.48	14.48 ± 3.02**
	ATDs	20.53 ± 2.54	19.68 ± 2.3	17.38 ± 3.07***	14.92 ± 2.48***
TSH	ML	11.68 ± 2.18	12.72 ± 1.28	13.4 ± 1.71	15.01 ± 2.17*
	MH	11.62 ± 0.92	13.33 ± 1.85*	13.69 ± 1.56*	16.77 ± 2.93**
	PL	9.66 ± 1.8	12.06 ± 1.97*	10.03 ± 1.06	12.39 ± 2.16*
	PH	11.29 ± 1.41	10.9 ± 1.48	14.1 ± 1.64**	14.15 ± 0.92***
	ATDs	11.01 ± 1.78	12.22 ± 1.83*	12.66 ± 2.23**	14.25 ± 2.54***
Weight	ML	237.67 ± 10.87	244.44 ± 16.44	232.98 ± 16.95	238.73 ± 7.8
	MH	228.51 ± 15.07	230.11 ± 21.67	227.62 ± 25.31	219.68 ± 15.93
	PL	230.79 ± 11.66	224.58 ± 18.48	210.3 ± 20.29*	199.9 ± 13.46***
	PH	244.77 ± 8.74	223.46 ± 11.67**	205.31 ± 15.27***	194.83 ± 15.18***
	Control 1	248.6 ± 12.38	280.88 ± 15.5*	287.28 ± 9.6**	319.25 ± 8.84***

The Wilcoxon rank-sum test was used to compare these parameters and values are expressed as the mean ± SD. Statistical difference from corresponding baseline expressed as *p* values: *p < 0 .05, **p < 0 .01, ***p < 0.001.

In the animal study, liver function-related indexes, such as ALT, GGT, TBIL, DBIL and total bile acid (TBA), increased in both ATDs groups (the MMI and PTU groups) compared with those in the control group, while nutrition-related indicators, such as ALB, decreased (Supplementary Table S4). In addition, the ALT level increased more in the PTU group than in the MMI group (*p* = 0.033), while the TBA increased more in the MMI group (*p* = 0.011), which is consistent with the findings that MMI always causes cholestatic liver injury and that PTU often causes hepatocyte liver injury (Wang et al., 2014).

### Gut Microbiome Profiles Among Groups

#### Sequencing Characteristics

From all 123 fecal samples, 9,249,423 high-quality sequences were selected based on barcode- and primer-sequence filtering, with 75,199 (ranging from 51,739 to 98,762) sequences per barcoded sample. A total of 3,338 OTUs were delineated at a 97% similarity level. The rarefaction curve of all samples reached a plateau (Supplementary Figure S6C), and the average Good’s coverage for these samples was greater than 99.3%, which indicated a satisfactory gut microbiota sequencing depth. Furthermore, we divided these samples into 24 subgroups according to the aims of the study. The specific information is shown in Supplementary Table S5. The aims were as follows:

## Part 1: To Further Investigate Whether Antithyroid Drugs Can Cause Changes in the Gut Microbiota Structure (Antithyroid Drugs Group vs. Control Group)

### Alpha and Beta Diversity

**FIGURE 4:**
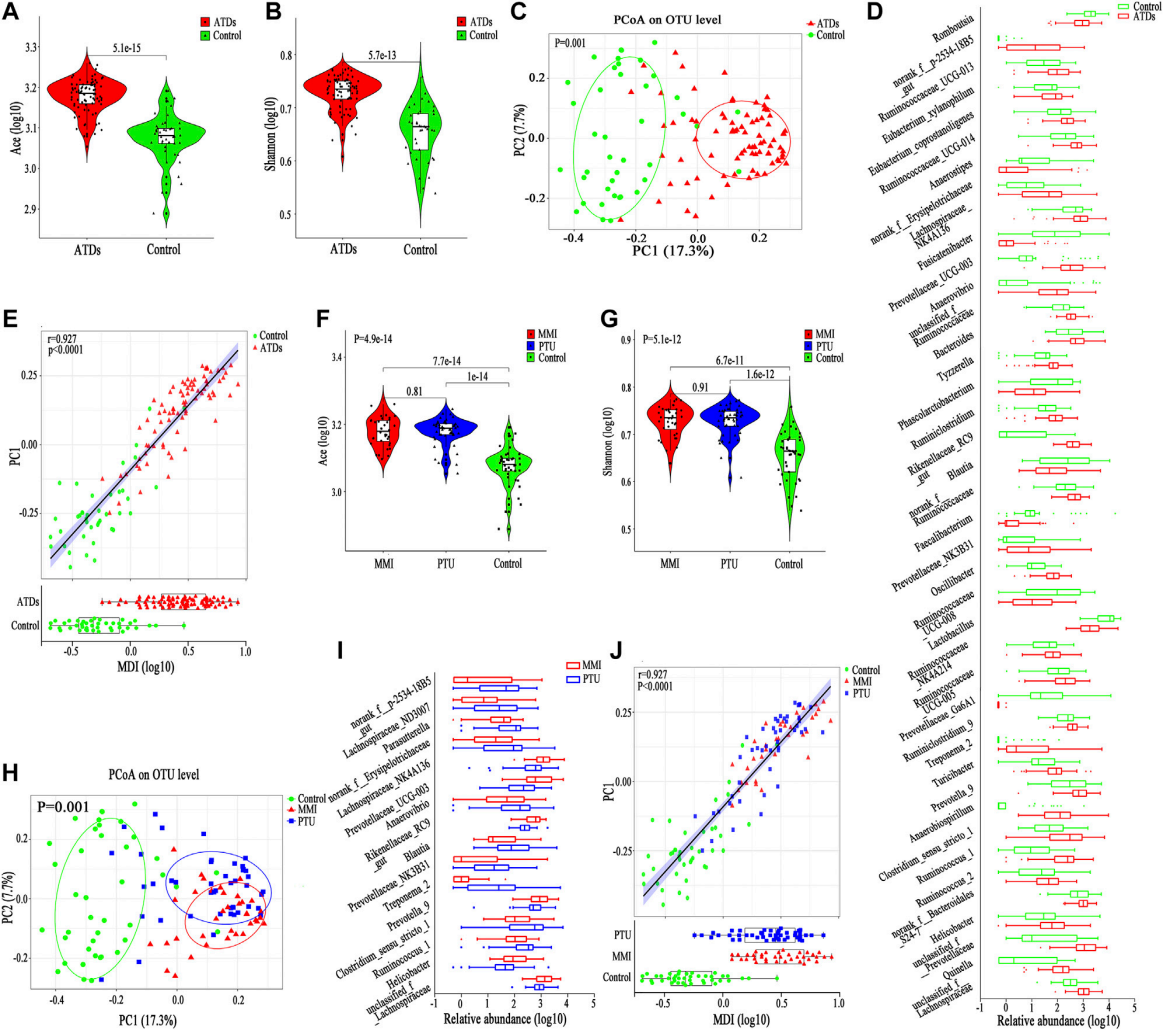
Change in basic parameters and the gut microbiota of SD rats. **(A,B)** Violin plot of the alpha diversity indexes for richness (Ace index) and evenness (Shannon index) of the gut microbiota among the ATDs and control groups. **(C)** PCoA based on the Bray-Curtis distance showing different clusters among the two groups (*p* = 0.001). **(D)** Comparisons of the relative abundances among differentially abundant genera by Mann-Whitney U-tests (*p* < 0.05). **(E)** Spearman correlation between the MDI and PC1 of the PCoA among the ATDs and control groups. **(F,G)** Violin plot of the alpha diversity Ace index and Shannon index of the gut microbiota among the MMI, PTU, and HC groups. **(H)** PCoA based on the Bray-Curtis distance showing different clusters among the MMI, PTU, and HC groups (*p* = 0.001). **(I)** Comparisons of the relative abundances among differentially abundant genera by Mann-Whitney U-tests (*p* < 0.05). **(J)** Spearman correlation between the MDI and PC1 of the PCoA among the ATDs and control groups.

The alpha diversity of the gut microbiota in the ATDs group (*n* = 82, including all the samples after ATDs administration) was significantly higher than that in the control group (*n* = 41, including all the samples before ATDs administration and the first three time points of Control one group) according to the Ace index (*p* = 5.1e-15), the Shannon index (*p* = 5.7e-13) and other diversity indexes (Figures 4A,B; Supplementary Table S5); the increased Ace and Shannon indexes after ATDs administration were consistent with the tendency for clinical variation, although no significant differences were found in the clinical study. PCoA revealed significant separation of the gut microbiota structure between the ATDs group and the control group (*p* = 0.001, Figure 4C), revealing a more obvious distinction than that in the clinical comparison of the Treat_GD and Init_GD groups. To show the specific changes caused by ATDs, we performed a differentially abundant species analysis.

### Differentially Abundant Species Analysis

We compared the gut microbiota compositions between the ATDs and control groups, and 60 differentially abundant species, representing four phyla, 15 families and 41 genera. At the phylum level, *Bacteroidetes*, *Proteobacteria* and *Spirochaetae* abundances were enriched in the ATDs group, whereas *Firmicutes* abundance was enriched in the control group (Supplementary Figure S6D). Interestingly, the *Firmicutes* proportion was increased, while the *Bacteroidetes* proportion was decreased in the clinical study, which may be due to the different microbiotas between humans and rats, to the different disease statuses, or to the fact that clinical cases are often accompanied by adjuvant drug interference. At the family level, the ATDs group-enriched species included mainly *Prevotellaceae* and *Ruminococcaceae*, while Lactobacillaceae and *Peptostreptococcaceae* were prevalent in the control group (Supplementary Figure S6D). Similar to at the phylum level, the shared differentially abundant families in humans and animals, *Ruminococcaceae* and *Peptostreptococcaceae*, showed different variation trends. *Lactobacillus*, *Romboutsia* and *Faecalibacterium* were enriched in the control group, while *Clostridium_sensu_stricto_1, Prevotellaceae_UCG-003* and *Oscillibacter* were enriched in the ATDs group (Figure 4D). The MDI also increased after ATDs intervention compared with that in the controls in the animal study, which was significantly correlated with PC1 (*r* = 0.927, *p* < 0.0001, Figure 4E), reflecting that the gut microbiota structure was disturbed in the ATDs group.

## Part 2: To Further Investigate Whether Different Antithyroid Drugs Cause Different Gut Microbiota Structure Changes (Methimazole vs. Propylthiouracil vs. Control; MMI_2 Vs. PTU_2, Including Subgroups With Specific Doses; MMI_3 Vs. PTU_3, Including Subgroups With Specific Doses; MMI_4 vs. PTU_4, Including Subgroups With Specific Doses)

### Alpha and Beta Diversity

Both the MMI and PTU groups showed higher Ace and Shannon indexes than the control group, but there was no significant difference between the two drug groups (Figures 4F,G). Interestingly, before the occurrence of hypothyroidism (at the 4th week and/or the 6th week), the Ace and Shannon indexes of the MMI group were higher than those of the PTU group, although they showed no significant difference, which is consistent with the changes observed in the clinical study after ATDs administration (Supplementary Table S5).

PCoA showed that both the MMI and PTU groups deviated from the control group. The MMI group was farther from the control group than the PTU group (*p* = 0.001, Figure 4H), which can also be seen from the separate comparison between MMI and PTU groups (*p* = 0.001, Supplementary Figure S6E). Interestingly, PCoA showed that different subgroups clustered slowly following the administration of ATDs, which may be because hypothyroidism aggravation plays a dominant role in the gut microbiota structure. On the other hand, before the occurrence of hypothyroidism (at the 4th week and/or the 6th week), subgroups within the MMI and PTU groups exhibited relatively greater separation from one another, reflecting the different effects caused by different ATDs on the gut microbiota (Supplementary Figures S6F–H).

### Differentially Abundant Species Analysis

To identify the specific structural changes associated with the administration of different ATDs, we compared the gut microbiota compositions in the MMI and PTU groups in the animal study, and 24 discriminative species, representing one phylum, seven families (Supplementary Figure S7A) and 16 genera (Figure 4I), were revealed with *p* < 0.05. At the phylum level, *Spirochaetae* was more prevalent in the PTU group than in the MMI group; the corresponding families Spirochaetaceae and Clostridiaceae*_1* also showed higher abundance in the PTU group, while Lachnospiraceae and *Rikenellaceae* were more prevalent in the MMI group (Supplementary Figure S7A). At the genus level, *Prevotella_9* and *Rikenellaceae_RC9* were enriched in the MMI group, while *Clostridium_sensu_stricto_1*, *Blautia*, and *Treponema_2* were more prevalent in the PTU group (Figure 4I). Although the microbiota in humans and rats differ and there were a few shared differentially abundant microbiota constituents, the MMI and PTU groups showed differentially abundant microbiota constituents in both the clinical and animal studies.

Similarly, the MDI significantly increased in both the MMI and PTU groups compared with that in the control group, and the MMI group exhibited a higher MDI than the PTU group, which is consistent with the clinical study results. Furthermore, the MDI was significantly correlated with PC1 (*r* = 0.927, *p* < 0.0001, Figure 4J), illustrating that gut microbiota dysbiosis occurred in both the MMI and PTU groups, while the MMI group showed more dysbiosis.

## Part 3: To Investigate Whether the Antithyroid Drugs-Induced Changes in the Gut Microbiota Structure Are Dose Dependent (ML_234 vs. MH_234; ML_2 vs. MH_2; ML_3 vs. MH_3; ML_4 vs. MH_4; PL_234 vs. PH_234; PL_2 vs. PH_2; PL_3 vs. PH_3; and PL_4 vs. PH_4)

### Alpha and Beta Diversity

**FIGURE 5:**
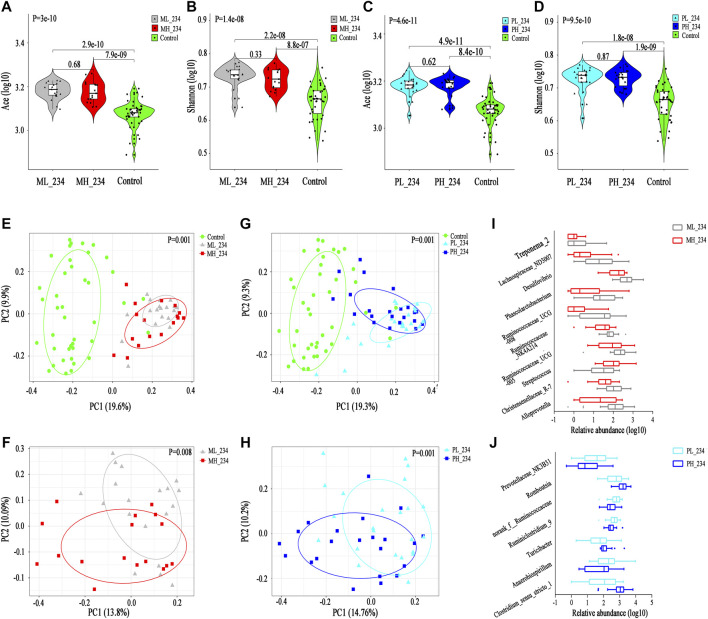
Change in gut microbiota structure in different dose groups among MMI and PTU groups. **(A,B)** Violin plot of the alpha diversity indexes for richness (Ace index) and evenness (Shannon index) of the gut microbiota among different MMI dose groups. **(C,D)** Violin plot of the alpha diversity indexes for richness (Ace index) and evenness (Shannon index) of the gut microbiota among different PTU dose groups. **(E,F)** PCoA based on the Bray-Curtis distance showing different clusters among ML_234, MH_234 and Control groups. **(G,H)** PCoA based on the Bray-Curtis distance showing different clusters among PL_234, PH_234 and Control groups. **(I)** Comparisons of the relative abundances among differentially abundant genera between the ML_234 and MH_234 groups by Mann‐Whitney U‐tests (*p* < 0.05). **(J)** Comparisons of the relative abundances among differentially abundant genera between the PL_234 and PH_234 groups by Mann‐Whitney U‐tests (*p* < 0.05).

Interestingly, in the MMI group, the alpha diversity index after ATDs administration was higher in the ML_234 group than in the MH_234 group, although there were no significant difference (Ace, *p* = 0.68; Shannon, *p* = 0.33, Figures 5A,B). In the PTU group, neither the Ace index (*p* = 0.62) nor the Shannon index (*p* = 0.87) showed a significant difference between the PL_234 and PH_234 groups, while the different indexes showed different variation trends (Figures 5C,D). For both the MMI and PTU groups, PCoA showed that subgroups corresponding to different doses deviated from one another (Figures 5E–H). Similar to the different ATD groups, the subgroups corresponding to different doses exhibited greater separation at the 4th and 6th weeks and had a tendency to cluster together as the ATDs intervention progressed (Supplementary Figures S7B–D). By the 10th week, there was no significant difference between the ML and MH groups (*p* = 0.081, Supplementary Figure S7D), which may be due to the dominant role of hypothyroidism.

### Differentially Abundant Species Analysis

In the MMI group, 17 discriminative species, representing two phyla, five families (*p* < 0.05, Supplementary Figure S7E) and 10 genera (*p* < 0.05, Figure 5I), were found between the ML and MH groups. Consistent with the alpha diversity result, the abundance of the most discriminative microbiota constituent was relatively higher in the ML group than in the MH group. For example, at the phylum level, both the differentially abundant phyla *Proteobacteria* and *Spirochaetae* were prevalent in the ML group. Similarly, at the family level, a higher abundance of *Desulfovibrionaceae* and Christensenellaceae was found in the ML group, while *Streptococcaceae* was more prevalent in the MH group, and the corresponding genera *Desulfovibrio* and *Streptococcus* showed the same trend*.* In the PTU group, ten discriminative features, representing three families (*p* < 0.05, Supplementary Figure S7F) and seven genera (*p* < 0.05, Figure 5J), were revealed between the PL and PH groups. At the family level, *Peptostreptococcaceae* and Clostridiaceae*_1* were abundant in the PH group, and *Succinivibrionaceae* was abundant in the PL group. The genera *Romboutsia* and *Clostridium_sensu_stricto_1* showed a higher abundance in the PH group than in the PL group, while *Anaerobiospirillum* showed a lower abundance.

## Part 4: To Further Investigate the Longitudinal Antithyroid Drugs-Induced Changes in the Gut Microbiota Structure at Different Time Points (ML_1 vs. ML_2 vs. ML_3 vs. ML_4; MH_1 vs. MH_2 vs. MH_3 vs. MH_4; PL_1 vs. PL_2 vs. PL_3 vs. PL_4; PH_1 vs. PH_2 vs. PH_3 vs. PH_4; all Include Comparisons Between One Another)

We next explored the longitudinal changes in the gut microbiota structure in four cohorts (the ML group, MH group, PL group and PH group) to eliminate the interference of different doses and drugs.

### Alpha and Beta Diversity

**FIGURE 6:**
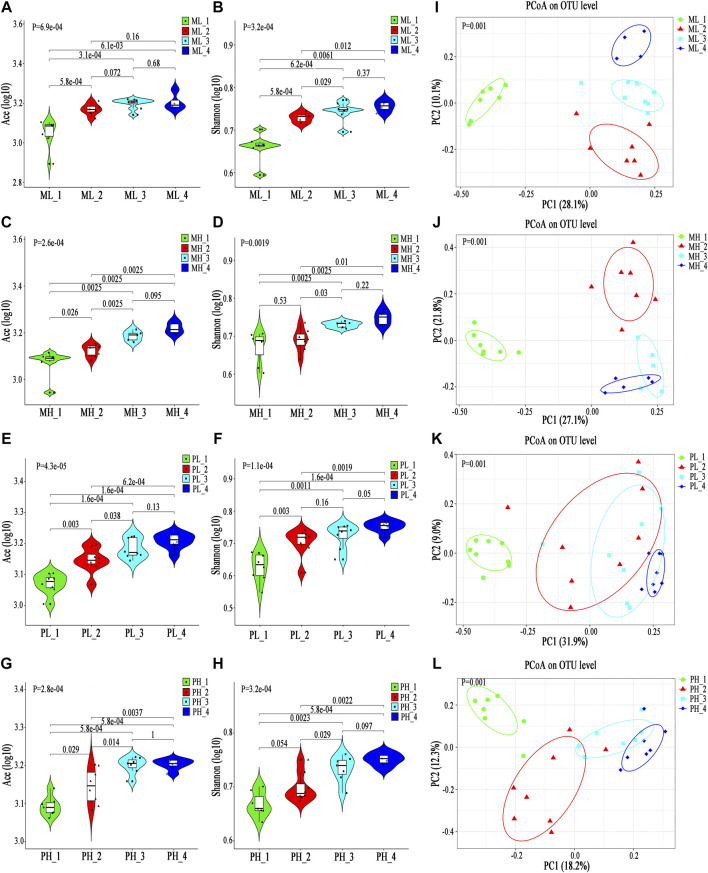
Longitudinal changes in the gut microbiota diversity and structure in SD rats. **(A-H)** Violin plot of the alpha diversity indexes for richness (Ace index) and evenness (Shannon index) of the gut microbiota longitudinal changes among the ML, MH, PL, and PH groups. **(I–L)** PCoA based on the Bray-Curtis distance showing different clusters among different subgroups.

Both the Ace and Shannon index gradually increased and then tended to stabilize along with ATDs application in all four cohorts (Figures 6A–H; Supplementary Table S5). Moreover, the PCoA results showed different clusters in the gut microbiota structure distribution at different time points among the four cohorts (Figures 6I–L); however, the distances between distributions decreased, which may be due to hypothyroidism progression.

### Differentially Abundant Species Analysis

Next, we performed a longitudinal analysis of differentially abundant microbiota constituents among the four cohorts, including a comprehensive analysis of the four time points and comparisons of each pair of time points. In the ML group, 63 discriminative species, representing four phyla, 17 families (*p* < 0.05, Supplementary Figure S8A) and 42 genera (*p* < 0.05, Supplementary Figure S9A), were revealed; in the MH group, 50 discriminative species, representing two phyla, 13 families (*p* < 0.05, Supplementary Figure S8B) and 35 genera (*p* < 0.05, Supplementary Figure S9B), were revealed; in the PL group, 56 discriminative species, representing four phyla, 17 families (*p* < 0.05, Supplementary Figure S8C) and 35 genera (*p* < 0.05, Supplementary Figure S9C), were revealed; and in the PH group, 57 discriminative species, representing five phyla, 16 families (*p* < 0.05, Supplementary Figure S8D) and 36 genera, were revealed (*p* < 0.05, Supplementary Figure S9D).

With ATDs administration, most microbiota constituents showed a longitudinal gradient change. At the phylum level, *Firmicutes* abundance significantly decreased and *Bacteroidetes* abundance increased in all cohorts except for the MH group, which showed the opposite trend, although there was no significant difference in the MH and PH groups. At the family level, Lactobacillaceae abundance decreased and *Ruminococcaceae* abundance increased with ATDs administration. Similarly, at the genus level, some genera varied in both the MMI and PTU groups, such as *Lactobacillus*, which decreased in abundance with ATDs use, while *unclassified_f_Prevotellaceae* abundance increased. However, some microbiota constituents showed different trends among different ATD treatments. For example, *Alloprevotella* abundance increased in the MMI group and decreased in the PTU group, although it varied in both drug administration groups. *Romboutsia* and Lachnospiraceae*_NK4A136* abundances varied mainly in the MMI group, while *Desulfovibrio* abundance varied mainly in the PTU group, which also reflects the different effects of different ATDs on the gut microbiota structure.

### Associations Between the Gut Microbiota and Basal Indicators

**FIGURE 7:**
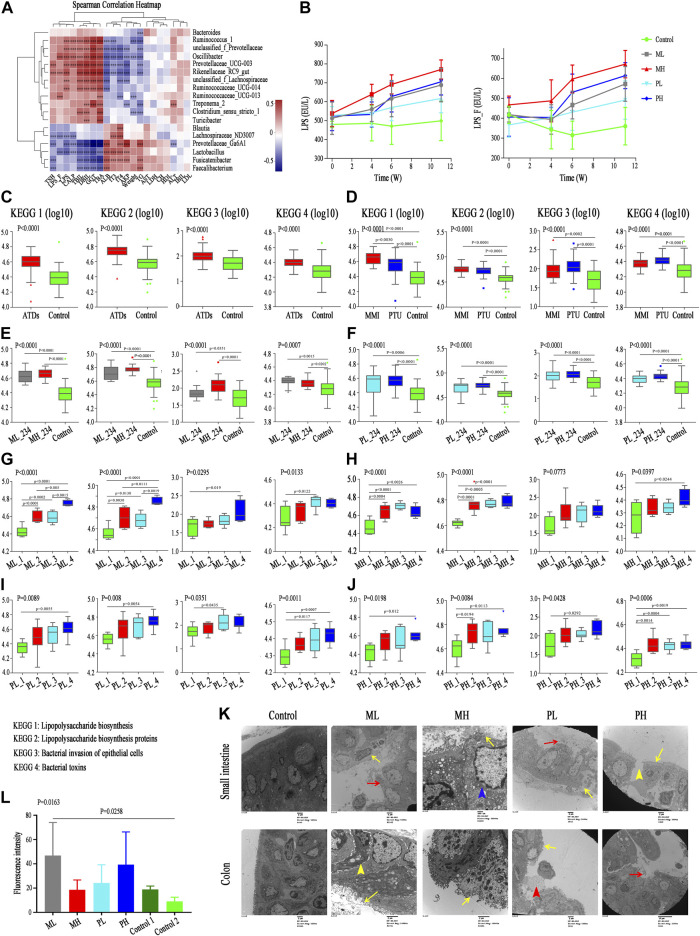
Correlation between differential microbiota and basal indicators, and change of KEGG and gut barrier related indexes. **(A)** Spearman correlation analysis of 21 basal indicators and 18 differential genera; color intensity represents the magnitude of correlation. Red, positive correlations; blue, negative correlations. *p value < 0.05; **p value < 0.01; ***p value < 0.001. **(B)** LPS concentration in serum (LPS) and fecal supernatant (LPS_F) among subgroups. **(C–J)** The change of pathway Lipopolysaccharide biosynthesis, Lipopolysaccharide biosynthesis proteins, Bacterial invasion of epithelial cells, and Bacterial toxins among groups with ATDs, different ATDs, different doses and different time points. **(K)** Transmission electron microscopy analysis of small intestine (upper) and colon (lower) among different subgroups. Yellow long arrow means epithelial villus shedding; red long arrow means intestinal epithelium shedding; yellow short arrow means cell shrinkage; blue arrow means cell swelling and red short arrow means cell debris. **(L)** Serum FITC-dextran levels among different groups.

The gut microbiota structure also changed after ATDs administration (including use or nonuse of drugs, different drugs, different doses and different time points). The correlation between the relative abundance of all differentially abundant genera (*n* = 49) and basal indicators (*n* = 21) was also analyzed by Spearman’s correlation in the animal study (Supplementary Figure S10). The abundances of ATDs group-enriched genera, such as *Prevotellaceae_UCG-003*, *Oscillibacter* and *Rikenellaceae_RC9*, were positively correlated with liver function-related indicators, such as GGT, TBIL, DBIL and TBA. However, these indicators were negatively correlated with the abundances of some control group-enriched genera, such as *Lactobacillus*, *Prevotellaceae_Ga6A1* and *Faecalibacterium* (Figure 7A). The correlation analysis showed that the change in the gut microbiota structure induced by ATDs may be correlated with liver function injury. Importantly, the microbiota constituents that were positively correlated with the liver function indexes were also positively correlated with LPS content in the fecal supernatant and serum (Figure 7A), and some genera, such as *Oscillibacter*, were enriched in an animal model of nonalcoholic fatty liver disease and positively correlated with plasma LPS content and intestinal permeability. Other clinical parameters, such as fT3 and fT4 levels, were positively correlated with *Lactobacillus*, *Fusicatenibacter* and *Faecalibacterium* and negatively correlated with *Oscillibacter*, *Prevotellaceae_UCG-003*, *Rikenellaceae_RC9*, etc. The TSH level showed an opposite relation with these microbiota constituents (Figure 7A).

### Functional Alterations in the Gut Microbiota

To characterize the functional alterations in the gut microbiota in the animal study, we also predicted the functional composition profiles using 16S rRNA sequencing data analyzed with PICRUSt. Similar to those in the clinical results, the metabolic pathways that were analyzed involved mainly pathways related to intestinal endotoxemia hypothesis.

Similar to the significant changes in the gut microbiota structure, the pathways of the Lipopolysaccharide biosynthesis, Lipopolysaccharide biosynthesis proteins, Bacterial invasion of epithelial cells, and Bacterial toxins were significantly enriched in the ATDs group compared with those in the control group (Figure 7C). Similarly, both the MMI and PTU groups showed increases in these four pathways compared with those in the control group, although no significant differences were found between the MMI and PTU groups. The Lipopolysaccharide biosynthesis and Lipopolysaccharide biosynthesis protein pathways were enhanced in the MMI group, which was consistent with the elevated LPS concentration in the fecal supernatant and serum in the MMI group. However, the Bacterial invasion of epithelial cells and Bacterial toxin pathways were higher in the PTU group than in the MMI group (Figure 7D). In the different dose groups, the four pathways were more enriched in the high-dose group than in the low-dose group, although most of them showed no significant difference (Figures 7E,F). Finally, the longitudinal changes among specific doses in the MMI and PTU groups were analyzed and showed an overall increasing trend following ATDs administration, which was also consistent with the changes in LPS content in the fecal supernatant and serum (Figures 7B,G–J).

The dysbiosis of the gut microbiota structure and function, accompanied by the increasing LPS concentration and corresponding changes in basal indicators, support our hypothesis. Gut microbiota composition alterations may be accompanied by intestinal barrier destruction, which allows microbiota-derived LPS to enter the blood and triggers a series of downstream inflammatory reactions and organ damage. Additionally, the fecal CALP concentration, which reflects the intestinal inflammatory response, is also an index reflecting intestinal permeability and intestinal barrier, increased with ATDs administration (Wilbrink et al., 2020) (Supplementary Table S4). Therefore, we further investigated relevant intestinal barrier indicators.

### Changes in the Intestinal Barrier Structure and Function

Transmission electron microscopy analysis of the control group showed that intestinal enterocytes presented regularly shaped mitochondria with intact cristae and microvilli that were thick, with lengths within the normal range (Figure 7K); colon enterocytes also presented regularly shaped mitochondria with intact cristae, and microvilli were neatly arranged but not as dense as those in the intestines. In the ML group, intestinal enterocytes showed only focal intestinal epithelial villus shedding and epithelial shedding; colon epithelial villus shedding was more severe than that in the intestines, some cells became pyknotic, and the intercellular space widened. MH group rats showed extensive epithelial villus shedding and mitochondrial swelling, and most of the colon epithelial villi were exfoliated. Similarly, in the PL group rats, intestinal enterocytes showed only focal intestinal epithelial villus shedding and epithelial shedding; colon enterocytes showed extensive exfoliated epithelial villi and some scattered cell debris. More severe damage appeared in the PH group, such as in the intestine, which showed a large area of epithelial villus exfoliation and cell rupture; most of the colon lacked a normal epithelial structure. These results all showed that 1) destruction was more severe in the colon than in the small intestine and that 2) the high-dose group experienced more severe effects than the low-dose group.

Consistent with the destruction of the intestinal barrier physical structure, ATDs group rats had significantly elevated serum FITC-dextran levels compared with those in the control group rats (Figure 7L). PH group rats showed higher serum FITC-dextran levels than those in the PL group rats, while interestingly, the ML group showed higher FITC-dextran levels than the MH group, which was consistent with the higher alpha diversity in the ML group than in the MH group.

## Discussion

In the present study, both clinical and animal studies showed that ATDs caused changes in the gut microbiota structure, and various liver function indexes, such as ALT and GGT, increased after ATDs administration. In addition, feces and serum LPS increased. The correlation between the disturbed gut microbiota structure, increased LPS level, and corresponding changes in clinical parameters indicate that the gut microbiota may mediate ATDs-induced liver injury through the intestinal endotoxemia hypothesis.

We focused mainly on the effect of ATDs on the gut microbiota structure. Although the differences in alpha diversity and beta diversity among subgroups in the clinical study were not always significant, they showed different variation tendencies. In the animal study, although the alpha diversity among different drug groups and most of the different dose groups showed no significant differences, nearly all the other alpha diversity and beta diversity indicators showed significant differences among the use or nonuse of ATDs, different ATDs, different doses and different time points, which reflect the fact that ATDs truly change the gut microbiota structure. The alpha diversity showed an increasing trend after ATDs administration in both the clinical and animal studies, which is contrary to the decrease of gut microbiota diversity caused by some drugs, such as antibiotics (Josefsdottir et al., 2017) and PPI (Jackson et al., 2016). This may due to intestinal bacterial overgrowth develops after recovery of hyperthyroidism in clinical study or hypothyroidism in animal study (Patil, 2014).

A differential gut microbiota analysis was also performed, and interestingly, the main changes in the microbiota in the clinical study differed from those in the animal study, as described above. For example, in the clinical study, ATDs-induced changes in the microbiota involved mainly *Faecalibacterium*, *Eubacterium_rectale*, *Romboutsia*, etc., while *Lactobacillus*, *Romboutsia*, *unclassified_f_Prevotellaceae*, etc., were changed primarily in the animal study. The abundance of some microbiota constituents, such as *Romboutsia,* increased in the clinical study but decreased in the animal study. These phenomena may be due to the differences between humans and rats. Furthermore, the clinical studies were based on the hyperthyroidism state in GD patients, whereas rats were evaluated under the ultimate hypothyroidism state. In addition, the clinical study also involved adjuvant drugs, such as hepatoprotective and leukogenic drugs. Though there were different gut microbiota constituents among clinical and animal studies, both showed that ATDs changed the gut microbiota and that different ATDs induced different gut microbiota structures. Furthermore, *Faecalibacterium*, which always reduced in some diseases such as Non-Alcoholic Fatty Liver Disease, showed low abundance both in clinical and animal experiments after ATDs administration (Zhou et al., 2018); *unclassified_f_*Lachnospiraceae, which was reported the key bacteria associated with inflammatory bowel disease patients or mouse model, also showed high abundance both in clinical and animal experiments after ATDs administration (Parada Venegas et al., 2019).

In addition, the gut microbiota and related metabolites all play important roles in maintaining homeostasis, and the same metabolites could be produced by different microbiotas. Recent studies have reported that SCFAs not only play an important role in regulating blood sugar and oxidizing energy supply but also are key in antipathogenic microorganisms for regulating intestinal microbiota balance, intestinal barrier function, systemic inflammation and hematopoiesis (Kim et al., 2014; Parada Venegas et al., 2019). In the present study, SCFAs-producing microbiota constituents, such as *Faecalibacterium*, *Ruminococcaceae*, *Lactobacillus* and *Blautia*, decreased after ATDs administration.

The destruction of intestinal barrier could permit the translocation of LPS produced by intestinal gram-negative bacteria across the intestinal barrier and triggers systemic inflammation (Zhou et al., 2017). Gut microbiota dysbiosis and increasing intestinal permeability have been shown to be associated with many diseases, such as autism spectrum disorders (Strati et al., 2017) and nonalcoholic steatohepatitis (NASH) (Shen et al., 2017). In addition, the gut microbiota and the intestinal barrier participate in the side effects of some drugs. For example, oral iron therapies are administered for the management or improvement of iron deficiency anemia (IDA), although this treatment is often accompanied by intestinal and liver injury, which may be caused by gut microbiota and intestinal barrier destruction (Fang et al., 2018). Similarly, cyclophosphamide treatment, which exerts antitumor effects, is accompanied by side effects, such as intestinal injury, and a calorie-restricted diet protects the gut microbiota and intestinal barrier from injury (Liu et al., 2019). Antibiotics disturb the microbiota structure and intestinal barrier, sometimes accompanied by myelosuppression; and gut microbiota reconstruction by fecal microbiota transplantation can partially restore hematopoiesis (Josefsdottir et al., 2017). In the present study, both intestinal barrier structure and function were destroyed, according to transmission electron microscopy and FITC-dextran tests, after ATDs administration. In addition, fecal CALP content, which reflects the intestinal inflammatory state, was also increased in both the clinical and animal studies. In addition to the SCFAs-producing microbiota mentioned above, the abundance of *Eubacterium_rectale,* which is also considered an intestinal barrier-protecting microbiota, decreased, while microbiota that destroy the intestinal barrier, such as *Dorea* and *Treponema_2*, increased, thus promoting intestinal barrier destruction (Del Chierico et al., 2016; Xie et al., 2016).

Another key molecule, LPS, which is believed to originate from the gram-negative gut microbiome, could leak into the systemic circulation and stimulate proinflammatory cytokine expression, inducing organ system injury. In addition, excessive LPS production could directly destroy the intestinal barrier, which in turn permits LPS to pass through the leaky gut (Cani et al., 2008). Animal studies revealed that LPS administration to mice via oral gavage induced a dramatic reduction in ZO-1 expression at 24 h, indicating intestinal barrier damage (Mouries et al., 2019). In the present study, the gut microbiota varied after ATDs exposure, and a characteristic change was the increase in the abundance of Enterobacteriaceae*,* which was thought to be the main LPS-producing microbiota (Vatanen et al., 2016) in the clinical study, especially in the MMI group. In an animal study, Enterobacteriaceae abundance also increased, although showing a lower abundance than that in the clinical study. In addition, analysis of microbiota functional alterations based on PICRUSt showed that the four LPS-related pathways were enriched after ATDs intervention compared with those in controls; the fecal and serum LPS levels in both the clinical and animal ATDs groups were higher than those in the control group, and the increase in LPS content was correlated with the change in blood indicators.

The bidirectional influence between the gut microbiota and drugs has long been studied. Broad-spectrum antibiotics induced gut microbiota dysbiosis which in turn is correlated with side effects, such as liver function abnormalities and hematopoietic depression, as mentioned above (Josefsdottir et al., 2017; Gong et al., 2018). Imhann et al. reported that long-term PPI use causes an increase in Enterobacteriaceae and a decrease in *Ruminococcaceae* abundances at the family level. These changes in symbiotic microbiota constituents increased susceptibility to *Clostridium difficile* infection (Jackson et al., 2016). ATDs also increased Enterobacteriaceae and decreased *Ruminococcaceae* abundances*,* and the increase in the Enterobacteriaceae abundance was correlated with intestinal barrier destruction, which could be reflected by the increase in fecal CALP content after the use of both PPIs and ATDs. Additionally, LPS and TLR-4 play important roles in the gastrointestinal toxicity effects of chemotherapeutic - irinotecan (Wardill et al., 2016) and the LPS and related pathways also showed higher levels after ATDs administration than in the controls in both the clinical and animal studies.

In summary, the ATDs caused changes in the gut microbiota structure, and the gut microbiota in turn was correlated with side effects. Not all of the side effects involved liver injury or hematopoietic depression; however, the drug-induced side effects were associated with LPS production and intestinal barrier destruction, which is consistent with our results.

## Conclusion

In the present study, we identified a close relationship among ATDs, gut microbiota dysbiosis, intestinal barrier destruction, LPS increase and liver injury. Gut microbiota dysbiosis was accompanied by intestinal barrier destruction, which induced increased LPS content entering the blood and triggering related liver injury. This study was the first to link the gut microbiota with ATDs-induced liver injury, which may provide new therapeutic targets and help reduce the liver injury of ATDs.

Inevitable limitations of our study must be mentioned. First, the clinical and animal experiments corresponded to two different thyroid function states, hyperthyroidism and hypothyroidism, which may lead to different gut microbiota changes. On the other hand, ATDs application must be accompanied by a change in thyroid function, and *in vitro* experiments would be unable to simulate the internal environment. Second, the clinical study involved the use of hepatoprotective drugs and leukogenic drugs, which may also play important roles in the gut microbiota structure. Third, this study did not conduct in-depth research on differentially abundant species, due mainly to the limitations of bacterial isolation culture techniques. Fourth, we performed a correlation analysis with no further intervention or mechanistic study, which need to be elucidated in the future.

## Data Availability Statement

The datasets presented in this study can be found in online repositories. The names of the repository/repositories and accession number can be found below: NCBI BioProject, accession no: PRJNA598313.

## Ethics Statement

The studies involving human participants were reviewed and approved by Ethics Committee of the First Affiliated Hospital of Harbin Medical University. The patients/participants provided their written informed consent to participate in this study. The animal study was reviewed and approved by Ethics Committee of the First Affiliated Hospital of Harbin Medical University.

## Author Contributions

JS, FZ, and YW designed and supervised the project. JS, BL, and JF collected the samples. JS, FZ, and BL performed the bioinformatics and statistical analyses and interpreted the data. JS and FZ drafted the manuscript. YW revised the manuscript for important content.

## Funding

This work was supported by the National Natural Science Foundation of China grants (81970466); 2017–2018 Special Fund for Scientific Research Transformation of Heilongjiang Academy of Medical Sciences (CR201803) and Project of scientific research and practical innovation for Postgraduates of Harbin Medical University (YJSKYCX2018-41HYD).

## Conflict of Interest

The authors declare that the research was conducted in the absence of any commercial or financial relationships that could be construed as a potential conflict of interest.
