# Supplementary material for: Gut Microbiota Participates in Antithyroid Drug Induced Liver Injury Through the Lipopolysaccharide Related Signaling Pathway
Source: Front Pharmacol. 2020 Dec 17;11:598170. doi: 10.3389/fphar.2020.598170 (PMC7774100; doi:10.3389/fphar.2020.598170)
Supplement: Supplementary file 1 [file datasheet1.doc]

**Supplementary information materials**

**Clinical study**

***Exclusion criteria***

Pregnancy; lactation; cigarette smoking; alcohol addiction; cardiovascular and metabolic diseases, such as hypertension, diabetes mellitus, hyperlipidemia and BMI>27; recent (<3 months prior) use of antibiotics, probiotics, prebiotics, symbiotics, hormonal medication, laxatives, proton pump inhibitors, insulin sensitizers or traditional Chinese medicine; known history of disease with an autoimmune component, such as multiple sclerosis, rheumatoid arthritis, irritable bowel syndrome, or IBD; and history of malignancy or any gastrointestinal tract surgery (e.g., gastrointestinal surgery, cholecystectomy or appendectomy).

***Blood and fecal sample collection***

All participants were examined in the morning after an overnight fast (≥8 h). Peripheral blood (15 mL) was collected from all subjects and stored in corresponding blood collection tubes at 4 °C for the detection of thyroid function indicators (free triiodothyronine, fT3; free thyroxine, fT4; and thyrotropin, TSH), thyroid autoantibody (antithyroglobulin antibody, Tg-Ab; and anti-thyroperoxidase antibody, TPO-Ab) examinations, and routine blood parameter, biochemical and serum LPS analyses. Concurrently, all subjects were provided Commode Specimen Collection Kits for feces collection, and samples were sent the to our laboratory on an ice pack within two hours. Each fecal sample was immediately divided into aliquots, frozen on dry ice, and stored at -80 °C for subsequent 16S rRNA gene and LPS analysis.

***Analysis of clinical parameters***

Serum fT3, fT4, and TSH levels were measured by a chemiluminescent immunoassay (Abbott Diagnostics, Tokyo, Japan). Tg-Ab and TPO-Ab were measured using a chemiluminescent immunoassay (Beckman Coulter, California, USA). Routine blood indexes were measured by flow cytometry (SYSMEX, Tokyo, Japan). Biochemical indexes were assessed using an automated biochemistry analyzer (Beckman Coulter, California, USA) and auxiliary reagents.

Serum alanine aminotransferase (ALT) and aspartate aminotransferase (AST) levels were measured by NADP-malate dehydrogenase ultraviolet spectrophotometry. Total protein (TP) levels were measured by the biuret method. Alkaline phosphatase (AKP) levels were measured by the nitrophenylphosphate-adenosine-5’-monophosphate (AMP) method. Total bile acid (TBA) levels were measured by enzymatic cycling methods. Total bilirubin (TBIL), direct bilirubin (DBIL), indirect bilirubin (IBIL) and γ-glutamyl transferase (GGT) levels were measured by the diazonium method.

***gDNA extraction***

DNA extraction was performed within one month after sample collection. Bacterial DNA was extracted from fecal samples at Novogene Bioinformatics Technology Co., Ltd. (Beijing, China) using a TIANGEN kit according to the manufacturer’s recommendations. DNA concentration and purity were monitored on 1% agarose gels. After concentration determination, DNA was diluted to 1 ng/µL using sterile water. The extracted DNA was stored at -20 °C. The bacterial DNA concentration was measured using a Nanodrop 2000 (Thermo Scientific, USA).

***Amplicon generation and purification***

Bacterial genomic DNA was amplified with the primers 341F (CCTAYGGGRBGCASCAG) and 806R (GGACTACNNGGGTATCTAAT), which are specific to the V3-V4 hypervariable regions of the 16S rRNA gene. All PCRs were carried out with Phusion® High-Fidelity PCR Master Mix (New England Biolabs, Ipswich, Massachusetts, USA). An equal volume of 1× loading buffer (containing SYBR green) was mixed with PCR products and subjected to electrophoresis on 2% agarose gels for detection. Samples with a bright, primary band between 400–450 bp were chosen for further experiments. The products of the same sample were combined and subjected to electrophoresis. DNA of the correct size was purified using a gel extraction kit (Qiagen, Hilden, Germany) and quantified using a Qubit instrument (Life Technologies, Carlsbad, CA).

***Library preparation and sequencing***

Sequencing libraries were generated using a TruSeq® DNA PCR-Free Sample Preparation Kit (Illumina, San Diego, CA, USA) following the manufacturer's recommendations, and index codes were added. Library quality was assessed using a Qubit@ 2.0 Fluorometer (Thermo Scientific, USA) and an Agilent Bioanalyzer 2100 system (Agilent Technologies, Santa Clara, CA). Finally, the library was sequenced on an Illumina HiSeq 2500 platform (Illumina), and 250-bp paired-end reads were generated.

***Paired-end read assembly and quality control***

Paired-end reads were assigned to samples based on their unique barcode and were truncated by trimming the barcode and primer sequence. Paired-end reads were merged using FLASH (version 1.2.7; San Jose, California, USA), a rapid and highly accurate analysis tool designed to merge paired-end reads when at least some of the reads overlap the reads generated from the opposite end of the same DNA fragment. The splicing sequences were termed raw tags.

Quality filtering of the raw tags was performed under specific filtering conditions to obtain high-quality clean tags according to the QIIME (version 1.7.0) quality control process. The tags were compared with a reference database (the Gold database) using the UCHIME algorithm to detect chimera sequences, and the chimera sequences were then removed. The effective tags were finally obtained.

***OTU clustering and species annotation***

Sequence analysis was performed using Uparse software (Uparse version 7.0.1001; Edgar, Tiburon, California, USA, http://drive5.com/uparse/). Sequences with ≥ 97% similarity were assigned to the same operational taxonomic unit (OTU). Representative sequences for each OTU were screened for further annotation. For each representative sequence, the SILVA128/16S database was used based on the Ribosomal Database Project (RDP) classifier (version 2.2) algorithm to annotate taxonomic information. To study the phylogenetic relationships between different OTUs and differences in dominant species in different samples (groups), we conducted multiple sequence alignment using MUSCLE software (version 3.8.31). OTU abundance information was normalized using the sequence number corresponding to the sample with the fewest sequences.

**Animal study**

***ATDs***

Both ATDs are clinical drugs. MMI (10 mg*50 tablets/3233799) was made at Merck KGaA, and PTU (50 mg*100 tablets/13120) was made at German Lomapharm Rudolf LohmannG. ATDs dose is calculated by weight conversion according to human dose.

***Sample collection and basic characteristics***

Before and after the 4th, 6th and 10th weeks (1 week before sacrifice to exclude the effect of FITC-dextran on other indexes) of ATDs administration, blood and fecal samples were routinely collected, and at the end of the study, various tissue samples and cecal contents were also collected. The specific collection method was as follows.

***Fecal sample and cecal contents*** Three fresh fecal pellets were individually placed in 2 mL sterile Eppendorf (EP) tubes by stimulating SD rats defecation. Additionally, after sacrifice at the 11th week, the cecal content was collected after the cecum was removed and weighed. The collection date and number were marked on the cap, and samples were snap frozen in liquid nitrogen and stored at -80 °C until further analysis (processing and analysis methods performed as described above).

***Blood sample*** Light isoflurane anesthesia was induced using a face mask, a precision vaporizer (Penlon PPV2) and a rodent ventilator (Hallowell EMC) when blood samples were collected. The induction time (time until loss of righting reflex) was 1-3 min. Then, the body weight was quickly measured, and a 0.05 mm microcapillary tube (length 75 mm, with an outer/inner diameter of 1.55/1.15 mm) was inserted at the inner canthus of the eye with a firm twist. As soon as blood appeared in the tube, 1.5-2.0 mL blood was collected, and then, a saline gauze sponge was gently placed over the eye to stop any residual bleeding. At the end of the study, each rat was anesthetized by intraperitoneal injection of 10% chloral hydrate, and blood was collected by aortaventralis puncture.

***Tissue samples*** When the rats were sacrificed, the thyroid, liver, spleen, cecum and total gastrointestinal tract were removed and placed in petri dishes containing 5 mL of saline and then weighed quickly (actual organ weight=total weight - petri dish weight). The tissue was collected for subsequent examination.

***Preparation of the fecal supernatant***

The fecal sample, weighing 0.1 g, was added to 0.9 mL of a precooled PBS solution according to the weight-volume ratio of 1:9. The samples was vortex oscillated until homogenization, liquid nitrogen freeze-thaw cycles were repeated three times, and a cell ultrasound breaker was used to break the cell wall for 10 seconds each time, with a total of six times. The homogenate was centrifuged (5000×g, 15 min), followed by filtration of the supernatant through a 0.22-μm filter and storage at ‒80 °C.

***Thyroid function, LPS and calprotectin indicator measurements***

All three indicators of rats were measured with corresponding ELISA kits (Shanghai Jiang Lai Biotechnology Co., Ltd., Shanghai, China) following the manufacturer's instructions. The thyroid function indicators we used were fT3, fT4 and TSH in the serum, serum and fecal supernatant LPS indicator (including human and animal samples) was measured, and fecal supernatant calprotectin was also tested. ***Biochemical and routine*** ***blood analysis of rats***

The biochemical index and routine blood analysis samples were sent to the animal laboratory of Harbin Medical University, and the biochemical indexes were determined by a corresponding kit using an automatic biochemical analyzer (SYSMEX, CHEMIX-180) and commercially available reagents (Sichuan Sinew Bio-technology Co., Ltd.). Routine blood parameter indexes were measured by flow cytometry (SYSMEX, Tokyo, Japan).

***Gut microbiota structure analysis***

Fecal and cecal content samples were collected and sent to Novogene Bioinformatics Technology Co., Ltd. (Beijing, China) for 16S rRNA gene sequencing, and the specific processing methods were the same as those used in the clinical study.

**Measurement of intestinal barrier function**

To evaluate intestinal barrier function, we analyzed the intestinal barrier by three methods. ***FITC-dextran*** At the end of the study, the rats were fasted overnight, and then, 4 kDa FITC-dextran (Sigma, 8 mg/100 g body weight) was administered by oral gavage 4 h before sacrifice. Each rat was anesthetized by intraperitoneal injection of 10% chloral hydrate, blood was collected by aortaventralis puncture, which was centrifuged at 3500 rpm at 4 °C for 10 min, and serum was collected. Then, FITC-dextran measurements were performed by a fluorescence spectrophotometer (F-7000, Hitachi, Japan) at an excitation wavelength of 490 nm and an emission wavelength of 520 nm, and all operations mentioned above were performed under light avoidance. ***Fecal calprotectin*** Fecal calprotectin was measured with the corresponding ELISA kit, as mentioned above. ***Transmission electron microscopy*** Transmission electron microscopy was mainly used for the detection of intestinal mucosal mechanical barrier, and observe the thinning and length of intestinal microvilli, the destruction of intestinal epithelial cells and the destruction of tight junctions between the epithelium. Three 1 cubic millimeter ileum and colon samples were immediately fixed in precooled 2.5% glutaraldehyde contained in a 1.5 mL EP tube at 4 ℃ overnight and then sent to the electron microscope room of Harbin Medical University. The samples were postfixed in 2.0% osmium tetroxide, dehydrated in a graded ethanol series and embedded in resin. Ultrathin sections (120 nm) stained with uranyl acetate and lead citrate were prepared for transmission electron microscopy (H7650, Hitachi, Tokyo, e Japan).

***Statistical analyses***

Alpha diversity was calculated to analyze the species diversity complexity in each sample based on 5 indexes, Good’s coverage, abundance-based coverage estimator (Ace), Chao, Shannon and Invsimpson. These indexes were calculated in our samples based on rarefied OTU counts. Beta diversity analysis was used to evaluate the similarities and differences in species complexity among the samples, and the beta diversity (Bray-Curtis distance) was calculated using QIIME software (version 1.7.0) based on the rarefied OTU counts and displayed using the weighted correlation network analysis (WGCNA) package, stats package and ggplot2 package in R software (version 2.15.3); and p value was calculate with anosim similarity analysis to evaluate whether the difference between groups (two or more groups) is significantly greater than that within groups. Permutational multivariate analysis of variance (PERMANOVA) of the distance matrices was implemented in the “vegan” package in R to determine whether the case/control status explained the variation in the gut microbial community composition.

Linear correlation analysis between the microbial dysbiosis index (MDI, the MDI is defined as the log of [total abundance in genera increased in disease group] over [total abundance of genera decreased in disease group]) or the *Firmicutes*/*Bacteroidetes* (F/B) ratio with PC1 was also performed using R software (version 2.15.3). A differential abundance analysis was performed using the Wilcoxon rank-sum test at the phylum, family and genus levels. The analyses were restricted to taxa with a prevalence>10% and a maximum proportion>0.002, and the taxon changes with a p value<0.05 were considered statistically significant (this method was used for all cases that are not otherwise described in the text). Predicted functional composition profiles were collapsed using PICRUSt into level 3 KEGG pathways based on 16S rRNA sequences. Correlations among the variables (clinical parameters, different microbiota, etc.) were computed using Spearman rank correlation, and the correlations (Student’s t test, p<0.05, r>0.3) are presented using a heatmap;

The gut microbiota metagenomes were imputed from 16S rRNA sequences using PICRUSt. The predicted functional composition profiles were collapsed into level 3 KEGG pathways, which represent pathways that are present in < 10% of the samples but not included in the comparison analysis. The correlations among the different species and different KEGG pathways were computed using Spearman rank correlation, and the correlations are presented using a heatmap.

**Results**

**Supplementary Tables**

Table S1 Clinical and demographic features of participants.

|  | **Group** | | | **Subgroup** | | | | | | | | **p Value** | | | | | | | |
| --- | --- | --- | --- | --- | --- | --- | --- | --- | --- | --- | --- | --- | --- | --- | --- | --- | --- | --- | --- |
| **Parameters** | **Init_GD** | **Treat_GD** | **HCs** | **MMI** | **PTU** | **M0** | **M2** | **M4** | **P0** | **P2** | **P4** | **p1** | **p2** | **p3** | **p4** | **p5** | **p6** | **p7** | **p8** |
| Age (year, mean±SD) | 37.7±10.89 | 37.45±11.24 | 36.58±9.06 | 36.1±12 | 39.31±10 | 36.1±12.15 | 36.1±12.15 | 36.1±12.15 | 39.3±9.5 | 37.82±10.97 | 40.22±9.58 | 0.838 | 0.925 | 0.594 | 0.422 | 0.289 | 1 | 0.819 | 0.409 |
| BMI (kg/m^2^, mean±SD) | 22.06±2.98 | 22.52±2.9 | 21.83±2.13 | 22.41±2.86 | 22.68±2.99 | 21.89±2.94 | 22.23±2.88 | 22.59±2.89 | 22.24±3.09 | 22.12±2.69 | 23.02±3.19 | 0.563 | 0.379 | 0.977 | 0.55 | 0.539 | 0.633 | 0.484 | 0.507 |
| fT3(pg/mL, mean±SD) | 18.38±7.31 | 6.19±2.44 | 3.05±0.31 | 5.87±2.59 | 6.63±2.18 | 16.87±7.78 | 7.24±2.39 | 4.49±2.03 | 19.88±6.67 | 7.59±1.98 | 6.04±2.14 | 0.000 | 0 | 0 | 0 | 0.095 | 0 | 0 | 0.078 |
| fT4 (ng/dL, mean±SD) | 3.84±1.27 | 1.89±0.79 | 1.09±0.11 | 1.75±0.8 | 2.08±0.76 | 3.62±1.37 | 2.09±0.84 | 1.42±0.61 | 4.05±1.15 | 2.54±0.82 | 1.8±0.58 | 0.000 | 0 | 0 | 0 | 0.044 | 0 | 0 | 0.133 |
| TSH (uIU/mL, mean±SD) | 0.003±0.004 | 0.137±0.75 | 1.99±0.973 | 0.22±0.982 | 0.02±0.03 | 0±0.003 | 0.03±0.032 | 0.41±1.379 | 0±0.005 | 0.03±0.024 | 0.02±0.033 | 0.000 | 0 | 0 | 0 | 0.583 | 0 | 0.042 | 0.86 |
| TGAb (IU/mL, mean±SD) | 228.48±331.5 | 113.87±224.08 | 0.82±1.01 | 94.07±213.65 | 141.19±238.82 | 228.68±348.89 | 99.3±223.64 | 88.85±208.87 | 228.28±322.25 | 151.5±283.43 | 134.89±215.81 | 0.000 | 0.012 | 0 | 0 | 0.304 | 0.118 | 0.357 | 0.534 |
| TPOAb (IU/mL, mean±SD) | 399.55±379 | 325.42±334.18 | 0.84±1.36 | 301.22±294.65 | 358.8±385.09 | 369.25±352.06 | 347.03±331.1 | 255.41±253.29 | 429.85±411.05 | 384.05±431.9 | 343.36±365.85 | 0.000 | 0.319 | 0 | 0 | 0.724 | 0.642 | 0.752 | 0.694 |
| LPS (EU/L, mean±SD) | 16±2.36 | 15.56±2.32 | 13.66±1.77 | 15.39±1.99 | 15.81±2.74 | 16.11±2.55 | 15.69±2.1 | 15.09±1.89 | 15.89±2.22 | 15.4±2.68 | 16.05±2.82 | 0 | 0.405 | 0 | 0 | 0.817 | 0.516 | 0.73 | 0.777 |
| LPS_F (EU/L, mean±SD) | 13.18±2.43 | 12.23±2.3 | 9.3±1.58 | 12.4±2.19 | 12±2.47 | 13.23±2.5 | 12.34±2.11 | 12.45±2.32 | 13.13±2.42 | 12.1±2.43 | 11.95±2.57 | 0 | 0.036 | 0 | 0 | 0.473 | 0.299 | 0.282 | 0.9 |
| CALP_F (ug/mL, mean±SD) | 180.09±25.97 | 169.67±26.77 | 139.2±19.97 | 169.91±25.9 | 169.33±28.4 | 183.26±28.22 | 172.9±29.72 | 166.91±21.79 | 176.91±23.81 | 162.26±27.48 | 173.66±28.84 | 0 | 0.067 | 0 | 0 | 0.78 | 0.163 | 0.29 | 0.447 |
| WBC (10^9^/L, mean±SD) | 5.97±1.22 | 5.83±1.57 | 5.99±1.04 | 6.01±1.83 | 5.6±1.12 | 6.07±1.47 | 5.84±1.85 | 6.17±1.83 | 5.87±0.93 | 5.78±1 | 5.49±1.2 | 0.547 | 0.532 | 0.758 | 0.42 | 0.568 | 0.805 | 0.732 | 0.787 |
| NEUT(10^9^/L, mean±SD) | 3.24±0.95 | 3.44±1.28 | 3.82±0.86 | 3.52±1.5 | 3.31±0.9 | 3.15±1.04 | 3.42±1.41 | 3.62±1.61 | 3.33±0.88 | 3.39±0.82 | 3.27±0.97 | 0.004 | 0.687 | 0.003 | 0.02 | 0.827 | 0.688 | 0.945 | 0.372 |
| LYMPH(10^9^/L, mean±SD) | 2.25±0.67 | 1.97±0.57 | 1.71±0.41 | 2.04±0.65 | 1.87±0.45 | 2.47±0.74 | 1.99±0.67 | 2.08±0.64 | 2.03±0.51 | 1.96±0.41 | 1.82±0.48 | 0.001 | 0.059 | 0 | 0.027 | 0.307 | 0.117 | 0.399 | 0.058 |
| MONO(10^9^/L, mean±SD) | 0.39±0.13 | 0.29±0.08 | 0.37±0.1 | 0.29±0.08 | 0.29±0.09 | 0.4±0.12 | 0.29±0.08 | 0.28±0.08 | 0.39±0.14 | 0.31±0.09 | 0.28±0.09 | 0.000 | 0 | 0.518 | 0 | 0.58 | 0.001 | 0.012 | 0.704 |
| EO(10^9^/L, mean±SD) | 0.08±0.07 | 0.12±0.09 | 0.08±0.06 | 0.12±0.09 | 0.11±0.08 | 0.08±0.07 | 0.13±0.1 | 0.12±0.09 | 0.08±0.06 | 0.11±0.09 | 0.1±0.08 | 0.009 | 0.02 | 0.867 | 0.013 | 0.342 | 0.043 | 0.749 | 0.349 |
| BASO(10^9^/L, mean±SD) | 0.01±0.01 | 0.01±0.01 | 0.02±0.02 | 0.01±0.02 | 0±0.01 | 0±0 | 0.01±0.01 | 0.02±0.02 | 0.01±0.01 | 0±0.01 | 0±0.01 | 0.000 | 0.203 | 0 | 0 | 0.007 | 0 | 0.231 | 0.05 |
| RBC(10^12^/L, mean±SD) | 4.92±0.46 | 5.03±0.47 | 4.56±0.36 | 5.01±0.45 | 5.06±0.5 | 4.82±0.41 | 5.03±0.51 | 4.98±0.39 | 5.01±0.5 | 5.11±0.55 | 5.04±0.49 | 0.000 | 0.153 | 0 | 0 | 0.752 | 0.175 | 0.83 | 0.102 |
| HCT(%, mean±SD) | 40.47±3.21 | 41.91±3.53 | 41.56±3.34 | 40.98±2.94 | 43.19±3.9 | 39.55±2.6 | 40.95±3.09 | 41.02±2.87 | 41.4±3.54 | 43.88±4.26 | 42.77±3.72 | 0.054 | 0.019 | 0.061 | 0.055 | 0.017 | 0.101 | 0.195 | 0.051 |
| MCV(fL, mean±SD) | 83.14±5.42 | 83.1±4.6 | 90.95±4.49 | 81.75±4.67 | 84.96±3.85 | 81.68±5.74 | 81.7±5.05 | 81.79±4.4 | 84.6±4.79 | 86.05±2.9 | 84.29±4.26 | 0.000 | 0.982 | 0 | 0 | 0.005 | 0.967 | 0.427 | 0.11 |
| MCH(pg, mean±SD) | 27.77±2.49 | 27.72±2.23 | 30.07±1.47 | 26.92±2.45 | 28.83±1.23 | 26.93±2.8 | 27±2.76 | 26.84±2.17 | 28.61±1.84 | 29.04±1.12 | 28.71±1.31 | 0.000 | 0.992 | 0 | 0 | 0 | 0.892 | 0.616 | 0.051 |
| MCHC(g/L, mean±SD) | 333.76±14.78 | 332.91±15.14 | 332.44±6.47 | 329.04±16.95 | 338.24±10.3 | 329.36±18.34 | 329.88±19.56 | 328.21±14.34 | 338.15±8.46 | 337.64±9.37 | 338.61±11.07 | 0.331 | 0.578 | 0.109 | 0.006 | 0.005 | 0.949 | 0.857 | 0.072 |
| PCT(%, mean±SD) | 0.27±0.06 | 0.28±0.06 | 0.16±0.04 | 0.29±0.07 | 0.26±0.04 | 0.29±0.06 | 0.29±0.08 | 0.29±0.06 | 0.25±0.06 | 0.26±0.04 | 0.26±0.05 | 0.000 | 0.789 | 0 | 0 | 0.023 | 0.913 | 0.982 | 0.154 |
| MPV(fL, mean±SD) | 10.53±0.84 | 10.37±0.67 | 6.25±1 | 10.41±0.7 | 10.32±0.65 | 10.63±0.87 | 10.55±0.65 | 10.28±0.73 | 10.44±0.83 | 10.24±0.66 | 10.37±0.66 | 0.000 | 0.412 | 0 | 0 | 0.519 | 0.432 | 0.834 | 0.57 |
| HGB(g/L, mean±SD) | 135.63±13.19 | 139.69±14.44 | 137.7±11.86 | 134.79±11.79 | 146.46±15.21 | 130.4±11.74 | 134.97±12.02 | 134.6±11.86 | 140.86±12.72 | 148.28±16.64 | 145.34±14.65 | 0.343 | 0.144 | 0.356 | 0.006 | 0.002 | 0.409 | 0.384 | 0.051 |
| PLT(10^9^/L, mean±SD) | 265.18±51.99 | 269.2±65.12 | 251.18±38.45 | 282.49±72.17 | 250.86±49.43 | 274.24±55.29 | 284±89.5 | 280.99±51.72 | 256.12±48.14 | 253±48.43 | 249.56±51.38 | 0.683 | 0.96 | 0.421 | 0.09 | 0.039 | 0.928 | 0.918 | 0.285 |
| ALT(U/L, mean±SD) | 30.31±13.62 | 44.64±66.45 | 15.46±7.46 | 47.6±46.11 | 40.55±87.93 | 33.99±15.89 | 45.19±32.76 | 50.02±57.26 | 26.64±9.99 | 65.27±142.49 | 25.44±12.43 | 0.000 | 0.858 | 0 | 0 | 0.062 | 0.593 | 0.811 | 0.213 |
| AST(U/L, mean±SD) | 28.01±7.97 | 35.17±39.23 | 18.52±4.62 | 35.9±21.24 | 34.15±55.75 | 28.68±7.24 | 35.11±15.5 | 36.7±26.16 | 27.34±8.78 | 50.35±90.47 | 24.26±5.5 | 0.000 | 0.687 | 0 | 0 | 0.025 | 0.47 | 0.445 | 0.534 |
| GGT(U/L, mean±SD) | 34.74±23.33 | 38.97±27.68 | 21.5±12.61 | 36.2±27.69 | 42.79±27.69 | 25.99±13.03 | 29.79±15.82 | 42.61±35.18 | 43.49±28.04 | 41.07±20.24 | 43.83±31.91 | 0.000 | 0.308 | 0 | 0 | 0.12 | 0.233 | 0.951 | 0.055 |
| TP(g/L, mean±SD) | 72.93±5.02 | 75.5±4.75 | 73.92±3.91 | 75.99±5.27 | 74.82±3.92 | 71.68±4.79 | 76.39±4.88 | 75.59±5.73 | 74.18±5.05 | 74.58±4.35 | 74.97±3.76 | 0.020 | 0.009 | 0.302 | 0.097 | 0.241 | 0.011 | 0.929 | 0.068 |
| ALB(g/L, mean±SD) | 42.68±3.76 | 43.92±2.84 | 45.16±2.83 | 44.31±3 | 43.39±2.55 | 42.84±3.52 | 44.51±3.1 | 44.11±2.97 | 42.53±4.08 | 43.24±2.35 | 43.49±2.72 | 0.001 | 0.044 | 0.001 | 0.037 | 0.13 | 0.317 | 0.392 | 0.49 |
| GLB(g/L, mean±SD) | 29.7±4.77 | 31.58±3.94 | 28.74±3.46 | 31.68±3.79 | 31.43±4.19 | 27.75±4.27 | 31.89±3.78 | 31.48±3.89 | 31.66±4.52 | 31.35±5.07 | 31.48±3.71 | 0.001 | 0.039 | 0.324 | 0.001 | 0.798 | 0.005 | 0.997 | 0.05 |
| AKP(U/L, mean±SD) | 150.25±67.93 | 179.7±83.69 | 66±21.09 | 167.98±96.26 | 195.87±60.31 | 136.65±75.28 | 153.77±93.93 | 182.19±98.85 | 163.86±58.45 | 186.08±56.89 | 201.86±63.14 | 0.000 | 0.04 | 0 | 0 | 0.025 | 0.202 | 0.09 | 0.058 |
| TBIL(umol/L, mean±SD) | 16.02±5.76 | 14.76±5.66 | 13.21±3.93 | 13.13±3.56 | 17±7.16 | 15.16±4.22 | 13.15±3.56 | 13.11±3.64 | 16.87±6.98 | 16.55±6.48 | 17.27±7.71 | 0.098 | 0.18 | 0.033 | 0.051 | 0.028 | 0.22 | 0.99 | 0.705 |
| DBIL(umol/L, mean±SD) | 3.33±1.21 | 2.88±1.06 | 2.32±0.64 | 2.71±0.87 | 3.12±1.26 | 3.14±0.85 | 2.63±0.92 | 2.8±0.83 | 3.52±1.48 | 3.07±0.95 | 3.14±1.45 | 0.000 | 0.051 | 0 | 0.012 | 0.217 | 0.168 | 0.627 | 0.588 |
| IBIL(umol/L, mean±SD) | 12.68±4.83 | 11.87±4.78 | 10.89±3.4 | 10.42±2.86 | 13.88±6.07 | 12.02±3.54 | 10.52±2.84 | 10.32±2.95 | 13.35±5.87 | 13.48±5.62 | 14.12±6.47 | 0.289 | 0.302 | 0.125 | 0.039 | 0.02 | 0.23 | 0.946 | 0.745 |
| TBA(umol/L, mean±SD) | 3.94±3.01 | 3.33±2.3 | 6.06±2.42 | 3.24±2.55 | 3.45±1.94 | 3.49±3.39 | 3.5±3.05 | 2.98±1.99 | 4.39±2.6 | 3.44±1.68 | 3.45±2.13 | 0.000 | 0.426 | 0 | 0 | 0.143 | 0.974 | 0.476 | 0.113 |
| PA(mg/L, mean±SD) | 176.87±54.13 | 204.2±52.64 | 226.28±43.62 | 187.23±35.48 | 227.62±63.2 | 160.45±33.74 | 183.7±36.01 | 190.75±35.51 | 193.3±65.64 | 223.45±62.85 | 230.17±65.1 | 0.000 | 0.002 | 0 | 0 | 0.018 | 0.024 | 0.107 | 0.151 |

The Wilcoxon rank-sum test was used to compare these parameters and values are expressed as the mean ± SD. p1: p Value of Init_GD vs. Treat_GD vs. HC; p2: p Value of Init_GD vs. Treat_GD; p3: p Value of Init_GD vs. HC; p4: p Value of MMI vs. PTU vs. HC; p5: p Value of MMI vs. PTU; p6: p Value of M0 vs. M2 vs. M4; p7: p Value of P0 vs. P2 vs. P4. p8: p Value of M0 vs. P0.

**Table S2 Comparison of phylotype coverage and diversity estimation of the 16S rRNA gene libraries at 97% similarity based on pyrosequencin**g analysis.

| **Sample/**  **Estimators** | **No. of Reads** | **No. of OTUs** | **Good’s Coverage (%)** | **Ace Mean** | **95% CI** | **Chao Mean** | **Shannon Mean** | **95% CI** | **Invsimpson Mean** |
| --- | --- | --- | --- | --- | --- | --- | --- | --- | --- |
| Init_GD | 3,156,080 | 2,580 | 99.73 | 644.61 | 590.66-698.56 | 627.3 | 3.72 | 3.55-3.9 | 19.31 |
| Treat_GD | 5,424,157 | 3,069 | 99.72 | 684.43 | 646.23-726.64 | 655.03 | 3.92 | 3.79-4.05 | 23.74 |
| HCs | 3,765,888 | 1,613 | 99.79 | 556.53 | 533.86-579.19 | 560.46 | 3.94 | 3.83-4.04 | 23.11 |
| **subgroup** |  |  |  |  |  |  |  |  |  |
| MMI | 3,169,026 | 2,417 | 99.7 | 722.96 | 687.11-778.07 | 681.86 | 3.84 | 3.65-4.04 | 22.86 |
| PTU | 2,255,131 | 2,094 | 99.75 | 631.29 | 552.12-710.47 | 618.03 | 4.02 | 3.85-4.19 | 24.94 |
| M0 | 1,605,009 | 1,926 | 99.68 | 694.84 | 645-744.69 | 683.83 | 3.73 | 3.56-3.91 | 18.72 |
| M2 | 1,597,235 | 2,104 | 99.68 | 749.18 | 674.88-823.47 | 717.2 | 3.86 | 3.62-4.1 | 21.48 |
| M4 | 1,571,791 | 1,840 | 99.72 | 696.74 | 644.32-749.16 | 646.51 | 3.83 | 3.5-4.15 | 24.24 |
| P0 | 1,551,071 | 1,802 | 99.76 | 594.38 | 498.75-690.01 | 590.77 | 3.71 | 3.39-4.03 | 19.89 |
| P2 | 826,214 | 1,863 | 99.71 | 736 | 537.03-934.97 | 746.21 | 4.22 | 3.9-4.55 | 28.35 |
| P4 | 1,428,917 | 1,352 | 99.78 | 567.31 | 521.89-612.72 | 539.7 | 3.9 | 3.7-4.09 | 22.85 |

Operational taxonomic units (OTUs) were defined at a 97% similarity level. The coverage percentage (Good’s) was calculated using Good’s method, and richness estimators (Chao and ACE) and diversity indices (Shannon and Simpson) were calculated using the Mothur program.

**Table S3 Common and specific differential microbiota among MMI, PTU and HC groups**

| **Additional common differential microbiota among MMI vs. HCs and PTU vs.HCs** |
| --- |
| **Phylum** |
| Actinobacteria |
| **Familly** |
| Lactobacillaceae, Porphyromonadaceae, Streptococcaceae, Ruminococcaceae, Acidaminococcaceae, Lachnospiraceae, Bifidobacteriaceae, Rikenellaceae |
| **Genus** |
| Lactobacillus, Anaerostipes, Faecalibacterium, Alistipes, Dorea, Ruminococcaceae_UCG-002, Fusicatenibacter, Bifidobacterium, Phascolarctobacterium, Eubacterium_eligens, Eubacterium_hallii, Lachnospiraceae_ND3007, Roseburia, Coprococcus_3, Streptococcus, Ruminococcaceae_NK4A214, unclassified_f_Lachnospiraceae, Parabacteroides |
| **Specific differential microbiota among MMI vs. HCs** |
| **Familly** |
| Christensenellaceae, Clostridiaceae_1 |
| **Genus** |
| Romboutsia, Holdemanella, Clostridium_sensu_stricto_1, Alloprevotella, Coprococcus_2, Christensenellaceae_R-7 |
| **Specific differential microbiota among PTU vs.HCs** |
| **Familly** |
| Erysipelotrichaceae, Prevotellaceae |
| **Genus** |
| Megasphaera, Eubacterium_coprostanoligenes, Ruminococcus_1 |

**Table S4 Basic parameters of SD rats.**

| **Parameters** | **Control** | **ATDs** | **MMI** | **PTU** | **MMI_2** | **PTU_2** | **MMI_3** | **PTU_3** | **MMI_4** | **PTU_4** | **ML_1** | **ML_2** | **ML_3** | **ML_4** | **MH_1** | **MH_2** | **MH_3** | **MH_4** | **PL_1** | **PL_2** | **PL_3** | **PL_4** | **PH_1** | **PH_2** | **PH_3** | **PH_4** | **ML_234** | **MH_234** | **PL_234** | **PH_234** | **p1** | **p2** | **p3** | **p4** | **p5** | **p6** | **p7** | **p8** |
| --- | --- | --- | --- | --- | --- | --- | --- | --- | --- | --- | --- | --- | --- | --- | --- | --- | --- | --- | --- | --- | --- | --- | --- | --- | --- | --- | --- | --- | --- | --- | --- | --- | --- | --- | --- | --- | --- | --- |
| Weight | 246.1±22.99 | 220.05±21.83 | 232.7±18.78 | 210.15±18.86 | 237.28±19.92 | 224.02±14.94 | 230.92±19.71 | 207.97±17.68 | 228.14±15.82 | 197.53±14.01 | 237.67±10.87 | 244.44±16.44 | 232.98±16.95 | 238.73±7.8 | 228.51±15.07 | 230.11±21.67 | 227.62±25.31 | 219.68±15.93 | 230.79±11.66 | 224.58±18.48 | 210.3±20.29 | 199.9±13.46 | 244.77±8.74 | 223.46±11.67 | 205.31±15.27 | 194.83±15.18 | 238.41±15.47 | 226.31±20.5 | 211.59±19.78 | 208.58±18.13 | 0 | 0 | 0.506 | 0.822 | 0.003 | 0 | 0.052 | 0.594 |
| fT3 (pg/ml) | 6.29±0.82 | 5.34±1 | 5.18±1.11 | 5.47±0.9 | 6.02±0.61 | 6.07±0.77 | 5.21±0.86 | 5.62±0.67 | 3.81±0.57 | 4.67±0.62 | 6.09±0.75 | 6.39±0.54 | 5.61±0.85 | 3.8±0.59 | 5.76±0.59 | 5.66±0.46 | 4.56±0.32 | 3.82±0.62 | 6.84±0.63 | 6.14±0.71 | 5.97±0.63 | 4.88±0.49 | 5.42±0.45 | 6±0.87 | 5.21±0.48 | 4.43±0.7 | 5.52±1.18 | 4.79±0.92 | 5.67±0.82 | 5.25±0.95 | 0 | 0.206 | 0 | 0 | 0 | 0.001 | 0.049 | 0.117 |
| TSH (uIU/ml) | 10.89±1.63 | 12.96±2.33 | 13.94±2.2 | 12.19±2.15 | 13.03±1.56 | 11.48±1.78 | 13.51±1.59 | 11.93±2.48 | 15.99±2.63 | 13.21±1.87 | 11.68±2.18 | 12.72±1.28 | 13.4±1.71 | 15.01±2.17 | 11.62±0.92 | 13.33±1.85 | 13.69±1.56 | 16.77±2.93 | 9.66±1.8 | 12.06±1.97 | 10.03±1.06 | 12.39±2.16 | 11.29±1.41 | 10.9±1.48 | 14.1±1.64 | 14.15±0.92 | 13.49±1.79 | 14.45±2.54 | 11.49±2.02 | 12.95±2.07 | 0 | 0 | 0.05 | 0.002 | 0.008 | 0 | 0.196 | 0.02 |
| fT4 (ng/dl) | 20.86±2.63 | 17.5±3.25 | 16.73±3.06 | 18.1±3.3 | 19±2.25 | 20.28±2.23 | 16.07±2.81 | 18.51±2.91 | 14.17±1.92 | 15.37±2.73 | 19.9±2.03 | 19.47±2.69 | 17.68±1.67 | 14.87±2.49 | 20.97±3.06 | 18.53±1.81 | 13.49±2.32 | 13.61±1.34 | 21.42±2.06 | 19.76±2.26 | 19.7±2.86 | 16.15±2.37 | 19.73±3.03 | 20.81±2.23 | 17.14±2.48 | 14.48±3.02 | 17.75±2.74 | 15.6±3.07 | 18.54±2.96 | 17.63±3.64 | 0 | 0.058 | 0.006 | 0 | 0.001 | 0.001 | 0.033 | 0.356 |
| LPS | 511.92±70.68 | 617.77±93.99 | 645.36±93.25 | 596.18±89.74 | 594.65±70.23 | 537.02±63.88 | 645.42±87.82 | 596.91±79.18 | 724.15±83.49 | 658.57±84.1 | 512.73±85.45 | 550.68±57.43 | 616.59±96.36 | 665.88±84.02 | 537.71±68.89 | 638.62±53.62 | 691.54±50.69 | 770.77±50.35 | 527.23±46.91 | 541.17±65.82 | 569.73±70.3 | 617.32±75.8 | 524.45±77.2 | 532.88±66.13 | 627.98±82.17 | 705.71±70.28 | 602.68±88.95 | 693.05±74.42 | 576.07±74.84 | 618.13±100.78 | 0 | 0.018 | 0.024 | 0 | 0.05 | 0 | 0.002 | 0.113 |
| LPS_F | 396.5±76.94 | 493.64±115.43 | 518.03±125.55 | 474.55±104.27 | 430.05±102.03 | 403.19±46.41 | 529.44±102.87 | 476.97±102.22 | 638.38±78.25 | 548.25±102.7 | 417.45±36.89 | 373.17±61.99 | 465.86±70.05 | 597.08±77.12 | 466.98±43.9 | 486.93±105.66 | 631.16±46.28 | 671.43±68.66 | 365.93±61.53 | 402.83±45.1 | 430.26±91.58 | 491.08±97.11 | 406.14±96.64 | 403.56±50.8 | 530.35±91.45 | 613.59±65.34 | 459.34±106.31 | 583.62±114.48 | 441.39±86.38 | 510.73±111.8 | 0 | 0.091 | 0 | 0 | 0.022 | 0 | 0.002 | 0.022 |
| CALP | 109.76±18.34 | 130.92±22.38 | 136.87±23.21 | 126.27±20.78 | 126.65±23.94 | 115.71±15.4 | 140.15±21.92 | 120.76±20.98 | 148.01±19.11 | 143.05±15.25 | 118.78±15.07 | 114.41±16.35 | 131.83±19.34 | 138.48±10.46 | 119.2±16.73 | 138.89±25 | 153.47±20.64 | 155.64±22.01 | 101.91±19.15 | 117.81±7.34 | 110.67±20.39 | 138.43±12.54 | 118.54±14.18 | 113.62±21.08 | 132.31±15.85 | 148.33±17.27 | 126.81±18.82 | 148.11±22.93 | 122.3±18.31 | 130.61±22.82 | 0 | 0.033 | 0.073 | 0.026 | 0.001 | 0.004 | 0.004 | 0.178 |
| GGT (U/L) | 1.8±0.78 | 4.29±1.67 | 4.06±1.24 | 4.48±1.94 | 3.79±1.05 | 3.06±0.68 | 3.85±0.99 | 3.87±1.13 | 4.78±1.64 | 6.6±1.68 | 2.14±0.38 | 4.57±0.54 | 4.13±1.13 | 5.75±1.71 | 1.29±0.49 | 3±0.82 | 3.4±0.55 | 4±1.23 | 2±0.76 | 2.75±0.71 | 4.25±1.17 | 6.63±1.77 | 1.57±0.79 | 3.38±0.52 | 3.43±0.98 | 6.57±1.72 | 4.63±1.21 | 3.41±0.94 | 4.54±2.04 | 4.41±1.87 | 0 | 0.235 | 0 | 0 | 0 | 0 | 0.002 | 0.82 |
| ALT (U/L) | 44±10.59 | 51.62±15.6 | 47.06±10.55 | 55.2±17.94 | 43.36±10.26 | 57.88±14.33 | 43.77±7.37 | 45.13±11.41 | 57.56±8.31 | 62.4±22.63 | 45.86±2.04 | 37.86±3.93 | 41.88±7.77 | 58.25±11.82 | 40.43±7.79 | 48.86±11.92 | 46.8±6.22 | 57±5.7 | 35.75±9.97 | 58.5±13.76 | 42±12.76 | 48.63±15.93 | 39.71±12.78 | 57.25±15.8 | 48.71±9.25 | 78.14±18.9 | 43.84±10.66 | 50.65±9.47 | 49.71±15.24 | 61.18±19.06 | 0.006 | 0.012 | 0.001 | 0.031 | 0.013 | 0 | 0.052 | 0.029 |
| AST (U/L) | 137.54±36.65 | 122.7±29.81 | 105.78±22.56 | 135.93±28.21 | 114.57±19.23 | 137.25±24.79 | 106.77±23.68 | 125.47±18.6 | 90.67±19.75 | 145±36.7 | 170.14±23.36 | 116.86±24.77 | 112.5±24.36 | 88±25.01 | 145.29±25.36 | 112.29±13.26 | 97.6±21.76 | 92.8±17.27 | 127±23.48 | 145.38±27.92 | 138±14.68 | 157.25±40.17 | 115.43±38.98 | 129.13±19.65 | 111.14±10.37 | 131±28.83 | 108.95±25.82 | 102.24±18.37 | 146.88±29.32 | 124±21.87 | 0.028 | 0 | 0 | 0.001 | 0.203 | 0.408 | 0.38 | 0.005 |
| AKP (U/L) | 128.41±33.28 | 90.99±38.45 | 86.11±32.6 | 94.8±42.43 | 67.43±17.11 | 95.5±40.5 | 81.92±20.59 | 88.67±25.23 | 121.22±38.87 | 100.2±57.63 | 156±20.98 | 65.71±13.76 | 81.5±21.84 | 137.75±41.07 | 117.14±14.36 | 69.14±20.92 | 82.6±20.89 | 108±35.57 | 147.25±33.24 | 84.75±29.3 | 87.75±26.95 | 74.5±16.18 | 125.43±14.15 | 106.25±48.91 | 89.71±25.21 | 129.57±74.55 | 87.53±35.94 | 84.53±29.44 | 82.33±24.41 | 108.41±53.24 | 0 | 0.313 | 0 | 0.004 | 0 | 0.375 | 0.787 | 0.044 |
| ALB (g/L) | 29.51±1.77 | 26.07±3.03 | 25.39±2.59 | 26.61±3.27 | 26.42±2.34 | 28.99±2.29 | 25.09±2.84 | 25.02±3.99 | 24.23±2.2 | 25.65±1.62 | 29.56±1.98 | 26.26±1.46 | 24.98±2.84 | 25.08±2.56 | 29.93±1.26 | 26.59±3.11 | 25.28±3.18 | 23.56±1.87 | 29.58±2.17 | 29.36±2.38 | 23.01±3.81 | 25.14±1.31 | 28.93±1.81 | 28.61±2.29 | 27.31±2.95 | 26.24±1.83 | 25.47±2.31 | 25.31±2.95 | 25.84±3.73 | 27.45±2.49 | 0 | 0.072 | 0.004 | 0.002 | 0 | 0.123 | 0.859 | 0.096 |
| LDH (mmol/L) | 1826.68±679.65 | 1752.51±534.68 | 1790.39±570.59 | 1722.87±509.23 | 1784.71±511.54 | 1565.5±555.23 | 1799.15±663.38 | 1987.8±444.02 | 1786.56±582.1 | 1625.8±438.7 | 2138.57±457.76 | 1871.14±542.82 | 1822.5±806.34 | 1667.75±540.03 | 2161.29±505.02 | 1698.29±504.85 | 1761.8±423.75 | 1881.6±658.44 | 1903.38±588.43 | 1369±657.1 | 2118±513.46 | 1634.75±577.97 | 1984.57±592.92 | 1762±374.92 | 1839±322.51 | 1615.57±243.11 | 1807.84±636.94 | 1770.88±504.99 | 1707.25±643.14 | 1739.91±319.49 | 0.544 | 0.574 | 0.635 | 0.399 | 0.083 | 0.4 | 0.849 | 0.827 |
| TBIL (umol/L) | 2.45±0.95 | 3.96±1.3 | 3.93±0.91 | 3.98±1.54 | 4.27±0.76 | 2.88±0.91 | 3.88±1.04 | 3.83±1.29 | 3.48±0.79 | 5.3±1.35 | 2.27±0.75 | 4.07±0.56 | 3.74±1.02 | 3.33±0.88 | 2.51±1.3 | 4.47±0.93 | 4.1±1.16 | 3.6±0.78 | 2.29±0.79 | 2.53±0.33 | 3.39±1.31 | 6.05±1.19 | 2.73±1.23 | 3.24±1.17 | 4.33±1.15 | 4.44±1.01 | 3.77±0.85 | 4.11±0.97 | 3.99±1.83 | 3.97±1.2 | 0 | 0.862 | 0.003 | 0.018 | 0 | 0.023 | 0.281 | 0.966 |
| DBIL (umol/L) | 1.51±0.78 | 2.99±0.95 | 2.91±0.67 | 3.06±1.12 | 2.96±0.49 | 2.25±0.66 | 2.95±0.95 | 3.21±0.97 | 2.78±0.44 | 3.76±1.15 | 1.31±0.75 | 2.73±0.31 | 3.04±0.77 | 2.88±0.49 | 1.69±0.79 | 3.2±0.55 | 2.8±1.27 | 2.7±0.44 | 1.64±0.6 | 2.03±0.34 | 2.8±0.95 | 4.4±1.11 | 1.61±1.08 | 2.48±0.84 | 3.69±0.81 | 3.03±0.7 | 2.89±0.57 | 2.94±0.79 | 3.08±1.31 | 3.04±0.91 | 0 | 0.468 | 0 | 0.015 | 0 | 0.001 | 0.841 | 0.909 |
| IBIL (umol/L) | 0.94±0.68 | 0.97±0.58 | 1.02±0.53 | 0.92±0.63 | 1.31±0.47 | 0.63±0.36 | 0.93±0.49 | 0.61±0.49 | 0.7±0.48 | 1.54±0.54 | 0.96±0.5 | 1.34±0.52 | 0.7±0.4 | 0.45±0.44 | 0.83±0.54 | 1.27±0.46 | 1.3±0.4 | 0.9±0.45 | 0.65±0.3 | 0.5±0.26 | 0.59±0.47 | 1.65±0.62 | 1.11±0.93 | 0.76±0.41 | 0.64±0.54 | 1.41±0.43 | 0.88±0.57 | 1.17±0.45 | 0.91±0.7 | 0.93±0.56 | 0.813 | 0.456 | 0.023 | 0.213 | 0 | 0.097 | 0.105 | 0.918 |
| TBA | 11.45±8.24 | 40.66±24.09 | 45.26±20.26 | 37.07±26.36 | 41.48±19.83 | 31.66±21.42 | 37.79±10.73 | 35.08±29.67 | 61.91±23.58 | 44.83±27.58 | 11.71±6.29 | 43.74±21.76 | 40.25±9.6 | 49.7±24 | 10.76±2.07 | 39.21±19.15 | 33.86±12.35 | 71.68±20.29 | 9.26±3.13 | 36.76±25.62 | 27.28±15.63 | 36.29±16.74 | 7.04±2.53 | 26.56±16.34 | 44±39.93 | 54.59±35.2 | 43.53±17.4 | 47.19±23.44 | 33.44±19.48 | 41.03±32.29 | 0 | 0.127 | 0.001 | 0 | 0.011 | 0.018 | 0.595 | 0.347 |
| CH (mmol/L) | 1.66±0.31 | 1.57±0.37 | 1.51±0.36 | 1.62±0.37 | 1.64±0.4 | 1.45±0.27 | 1.39±0.3 | 1.62±0.33 | 1.46±0.36 | 1.82±0.42 | 1.7±0.32 | 1.52±0.31 | 1.35±0.27 | 1.7±0.2 | 1.54±0.24 | 1.76±0.46 | 1.46±0.36 | 1.27±0.36 | 1.76±0.27 | 1.39±0.33 | 1.51±0.28 | 1.52±0.27 | 1.76±0.25 | 1.5±0.22 | 1.74±0.37 | 2.16±0.27 | 1.48±0.3 | 1.53±0.43 | 1.47±0.29 | 1.79±0.39 | 0.195 | 0.156 | 0.106 | 0.182 | 0.1 | 0.001 | 0.722 | 0.003 |
| TG (mmol/L) | 0.67±0.21 | 0.41±0.13 | 0.48±0.16 | 0.36±0.06 | 0.47±0.15 | 0.36±0.06 | 0.44±0.14 | 0.37±0.07 | 0.54±0.2 | 0.35±0.06 | 0.7±0.22 | 0.48±0.15 | 0.46±0.09 | 0.65±0.25 | 0.55±0.21 | 0.45±0.17 | 0.41±0.22 | 0.45±0.11 | 0.76±0.18 | 0.36±0.07 | 0.36±0.07 | 0.37±0.06 | 0.76±0.25 | 0.36±0.05 | 0.38±0.07 | 0.33±0.06 | 0.51±0.16 | 0.44±0.16 | 0.37±0.06 | 0.35±0.06 | 0 | 0 | 0.041 | 0.632 | 0 | 0 | 0.221 | 0.512 |
| HDL (mmol/L) | 0.86±0.14 | 0.85±0.21 | 0.86±0.22 | 0.84±0.21 | 0.89±0.23 | 0.79±0.2 | 0.81±0.24 | 0.78±0.18 | 0.9±0.18 | 0.94±0.22 | 0.95±0.13 | 0.85±0.21 | 0.71±0.14 | 0.97±0.17 | 0.85±0.09 | 0.92±0.26 | 0.96±0.3 | 0.85±0.19 | 0.9±0.11 | 0.78±0.26 | 0.75±0.2 | 0.8±0.2 | 0.82±0.13 | 0.8±0.13 | 0.82±0.16 | 1.09±0.14 | 0.82±0.2 | 0.91±0.24 | 0.78±0.21 | 0.9±0.19 | 0.619 | 0.57 | 0.037 | 0.769 | 0.479 | 0.001 | 0.21 | 0.041 |
| LDL (mmol/L) | 0.29±0.06 | 0.35±0.31 | 0.41±0.46 | 0.3±0.07 | 0.57±0.71 | 0.31±0.06 | 0.34±0.08 | 0.33±0.08 | 0.27±0.11 | 0.27±0.05 | 0.33±0.06 | 0.36±0.08 | 0.34±0.08 | 0.33±0.12 | 0.27±0.05 | 0.78±0.99 | 0.34±0.08 | 0.23±0.09 | 0.31±0.06 | 0.29±0.06 | 0.36±0.09 | 0.24±0.05 | 0.28±0.04 | 0.33±0.04 | 0.3±0.05 | 0.3±0.04 | 0.35±0.09 | 0.49±0.66 | 0.3±0.08 | 0.31±0.04 | 0.207 | 0.113 | 0.865 | 0.256 | 0.02 | 0.145 | 0.364 | 0.473 |

p1: p Value of Control vs. ATDs group; p2: p Value of MMI vs. PTU group; p3: p Value of ML_1 vs. ML_2 vs. ML_3 vs. ML_4; p4: p Value of MH_1 vs. MH_2 vs. MH_3 vs. MH_4; p5: p Value of PL_1 vs. PL_2 vs. PL_3 vs. PL_4; p6: p Value of PL_1 vs. PL_2 vs. PL_3 vs. PL_4; p7: p Value of ML_234 vs. MH_234; p8: p Value of PL_234 vs. PH_234.

**Table S5 Comparison of phylotype coverage and diversity estimation of the 16S rRNA ge**ne libraries for individuals at 97% similarity from pyrosequencing analysis.

| **Sample/**  **Estimators** | **No. of Reads** | **No. of OTUs** | **Good’s Coverage (%)** | **Ace Mean** | **95% CI** | **Chao Mean** | **Shannon Mean** | **95% CI** | **Invsimpson Mean** |
| --- | --- | --- | --- | --- | --- | --- | --- | --- | --- |
| **Group** |  |  |  |  |  |  |  |  |  |
| Control | 2,834,753 | 2,584 | 99.37 | 1,198.70 | 1152.55-1244.85 | 1,219.90 | 4.53 | 4.38-4.68 | 29.58 |
| ATDs | 6,414,670 | 3,247 | 99.26 | 1,521.62 | 1491.11-1552.14 | 1,546.57 | 5.37 | 5.28-5.45 | 78.29 |
| subgroup |  |  |  |  |  |  |  |  |  |
| MMI | 2,907,460 | 2,980 | 99.26 | 1,520.70 | 1472-1569.4 | 1,546.91 | 5.39 | 5.27-5.51 | 78.71 |
| PTU | 3,507,210 | 2,926 | 99.26 | 1,522.35 | 1481.83-1562.86 | 1,546.31 | 5.35 | 5.22-5.47 | 77.95 |
| subgroup |  |  |  |  |  |  |  |  |  |
| ML_234 | 1,554,934 | 2,829 | 99.26 | 1,549.58 | 1491.13-1608.02 | 1,579.47 | 5.52 | 5.4-5.64 | 90.43 |
| MH_234 | 1,352,526 | 2,496 | 99.27 | 1,488.42 | 1404.45-1572.4 | 1,510.52 | 5.25 | 5.04-5.45 | 65.61 |
| PL_234 | 1,845,275 | 2599 | 99.27 | 1,519.01 | 1460.43-1577.59 | 1,546.57 | 5.35 | 5.17-5.54 | 79.45 |
| PH_234 | 1,661,935 | 2767 | 99.25 | 1,525.99 | 1465.23-1586.74 | 1,546.03 | 5.34 | 5.17-5.5 | 76.33 |
| subgroup |  |  |  |  |  |  |  |  |  |
| ML_1 | 418,962 | 1,670 | 99.41 | 1,129.37 | 971.01-1287.73 | 1,154.36 | 4.58 | 4.28-4.89 | 28.4 |
| ML_2 | 561,679 | 2,113 | 99.27 | 1,478.26 | 1389.17-1567.34 | 1,510.08 | 5.36 | 5.23-5.48 | 71.32 |
| ML_3 | 679,969 | 2,202 | 99.25 | 1,581.02 | 1501.73-1660.32 | 1,611.12 | 5.56 | 5.33-5.79 | 94.1 |
| ML_4 | 313,286 | 2,208 | 99.26 | 1,611.50 | 1340.25-1882.74 | 1,637.60 | 5.71 | 5.41-6 | 116.54 |
| MH_1 | 431,662 | 1,739 | 99.38 | 1,192.11 | 1060.86-1323.37 | 1,221.59 | 4.71 | 4.3-5.12 | 34.12 |
| MH_2 | 488,529 | 1,962 | 99.31 | 1,331.30 | 1257.35-1405.26 | 1,349.17 | 4.91 | 4.58-5.23 | 43.76 |
| MH_3 | 431,716 | 1,989 | 99.25 | 1,538.15 | 1441.76-1634.54 | 1,570.66 | 5.38 | 5.25-5.5 | 67.89 |
| MH_4 | 432,281 | 2,102 | 99.22 | 1,658.66 | 1543.69-1773.62 | 1,676.27 | 5.6 | 5.31-5.9 | 93.92 |
| PL_1 | 548,696 | 1,750 | 99.37 | 1,180.26 | 1108.54-1251.99 | 1,213.98 | 4.26 | 3.91-4.61 | 19.44 |
| PL_2 | 472,419 | 2,049 | 99.3 | 1,405.11 | 1298.52-1511.69 | 1,433.44 | 5.09 | 4.70-5.47 | 55.94 |
| PL_3 | 706,948 | 2,171 | 99.26 | 1,532.65 | 1438.44-1626.86 | 1,561.58 | 5.32 | 4.95-5.69 | 73.47 |
| PL_4 | 665,908 | 2,218 | 99.25 | 1,619.28 | 1545.67-1692.89 | 1,644.69 | 5.66 | 5.47-5.85 | 108.93 |
| PH_1 | 478,352 | 1,819 | 99.35 | 1,240.47 | 1171.24-1309.7 | 1,257.29 | 4.65 | 4.42-4.88 | 32.17 |
| PH_2 | 517,730 | 2,192 | 99.27 | 1,404.76 | 1279.58-1529.95 | 1,416.95 | 5 | 4.74-5.27 | 52.06 |
| PH_3 | 595,265 | 2,270 | 99.23 | 1,590.28 | 1517.2-1663.36 | 1,610.77 | 5.42 | 5.14-5.69 | 80.9 |
| PH_4 | 548,940 | 2,163 | 99.26 | 1,600.24 | 1554.33-1646.14 | 1,628.80 | 5.64 | 5.5-5.77 | 99.49 |

Operational taxonomic units (OTUs) were defined at a 97% similarity level. The coverage percentage (Good’s) was calculated using Good’s method, richness estimators (Chao and ACE) and diversity indices (Shannon and Simpson) were calculated using the Mothur program.

**Supplementary Figures**

**Fig. S1 Flowchart illustrating recruitment of GD patients and healthy controls for the study.**


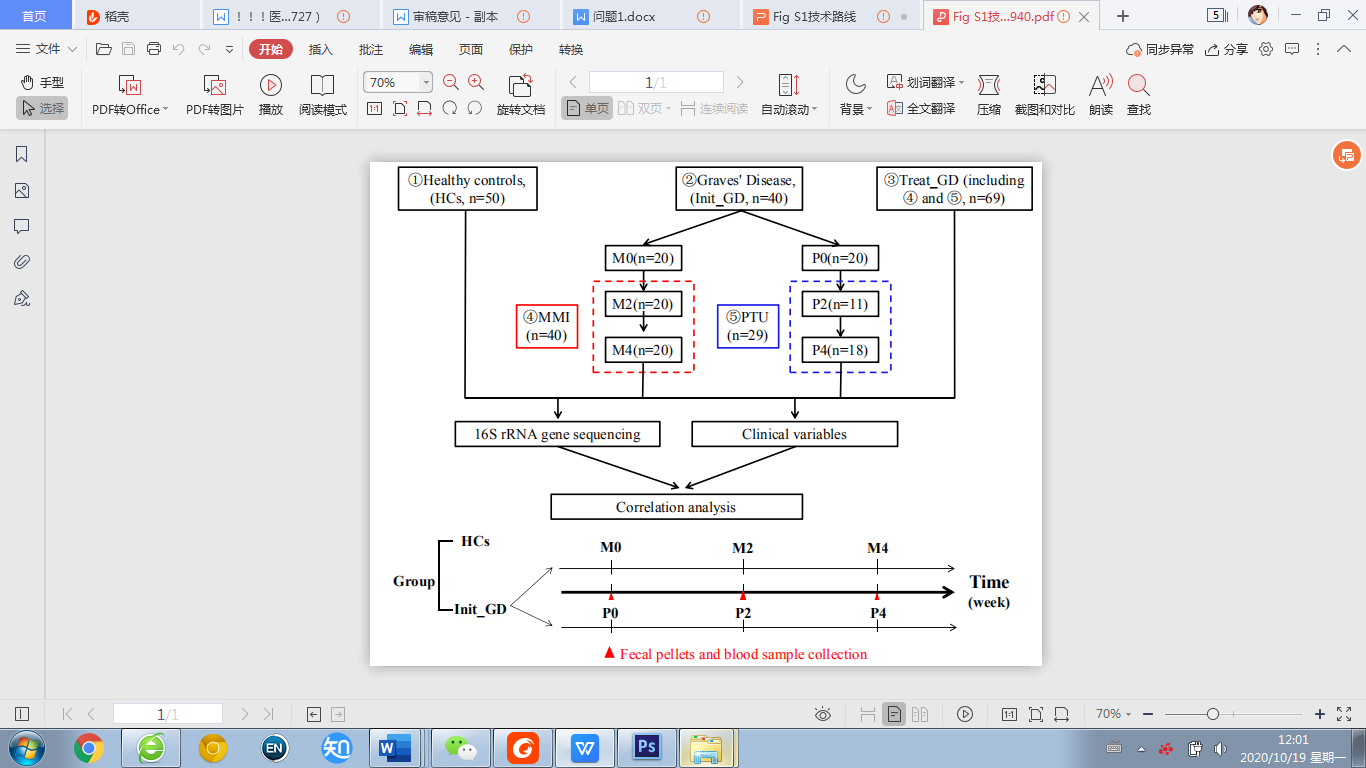


We recruited a total of 40 Init_GD patients (② Init_GD group), which were randomly divided into M0 (n=20) and P0 (n=20) according to the drug (i.e., MMI or PTU) they received and were followed up every two weeks during the first month (M2, n=20; M4, n=20; P2, n=11; and P4, n=18). After they took ATDs, they were divided into the ④ MMI group and the ⑤ PTU group, which together formed the ③ Treat_GD group. In addition, 50 age-, sex- and BMI-matched HCs (①) were included. The gut microbiota and related hematological indicators were measured and analyzed in all participants, and correlation analysis was then performed.

**Fig. S2 Animal study design and drug administration and sample collection timeline.**


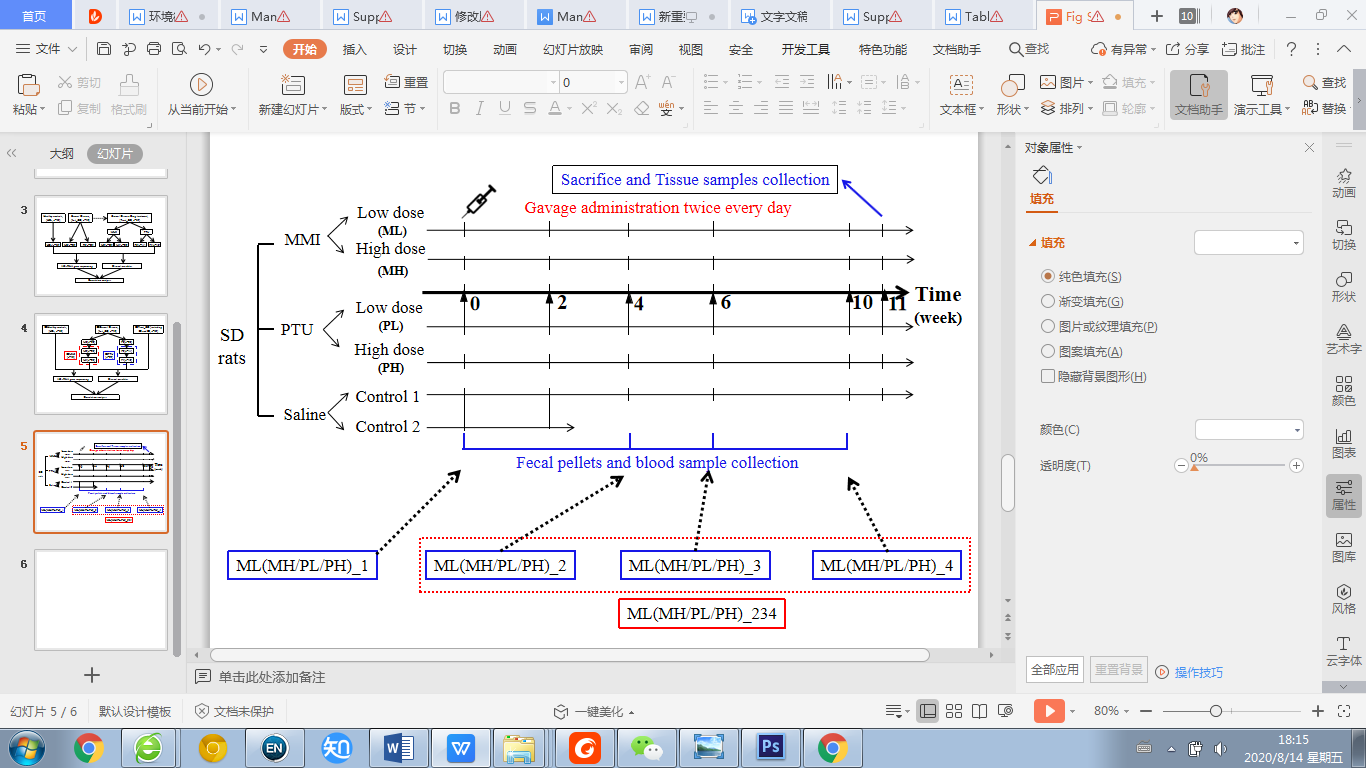


Rats were randomly allotted to 6 groups, and each received daily doses of 1.5 mg/kg/day MMI (ML group), 2.5 mg/kg/day MMI (MH group), 7.5 mg/kg/day PTU (PL group) or 12.5 mg/kg/day PTU (PH group) by oral gavage. The two control groups (n=4 for control 1; n=5 for control 2) received the same doses of vehicle (ordinary drinking water) every day. At the 2nd week after ATDs administration, thyroid volume was observed; fecal and blood samples were collected at the 4th, 6th, and 10th week after ATDs administration; and tissue and cecal samples were collected when rats were sacrificed at the 11th week. According to different time points of sample collection, we named the group as shown above, the first time of sample collection were classificated Control group; and MMI group including ML_234 and MH_234, PTU group including PL_234 and PH_234

**Fig. S3 Shift in the** **gut microbiota diversity and structure.**


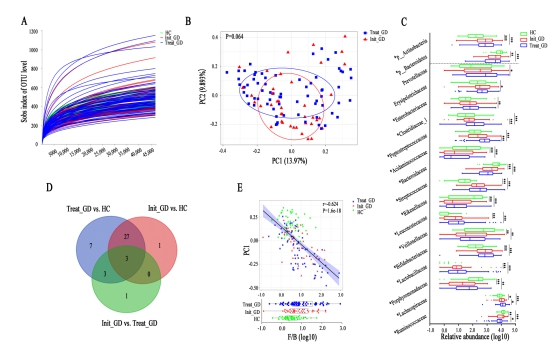


1. Rarefaction curves for each sample are nearly smooth, with a sufficient amount of sequencing data and few new undetected genes. (B) PCoA based the on Bray-Curtis distance showing different clusters among the Init_GD and Treat_GD groups (p=0.064). (C) Comparisons of the relative abundances of differentially abundant phyla and families (separated by dotted line) among the Init_GD, Treat_GD and HC groups by Kruskal-Wallis H test (p<0.05). (D) Venn diagram demonstrating the number of shared and specific differentially abundant genera of every pair of groups among the Init_GD, Treat_GD and HC groups. (E) Spearman correlation between the F/B ratio and PC1 of the PCoA. ^*^p indicates a p value <0.05 among the three groups, p^*^ indicates a p value <0.05 between each pair of groups. *, p value <0.05; **, p value <0.01; ***, and p value <0.001.

**Fig. S4 Change of gut microbiota structure among different drug and different time points groups.**


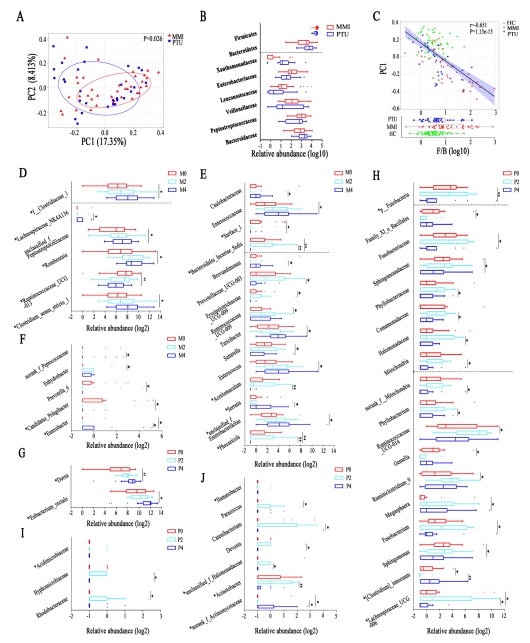


1. PCoA based on the Bray-Curtis distance showing different clusters among the MMI and PTU groups (p=0.026). (B) Comparisons of the relative abundances of differentially abundant phyla and families (separated by dotted line) among the MMI and PTU groups by Mann-Whitney U-tests (p<0.05). (C) Spearman correlation between the F/B ratio and PC1 of the PCoA. (D) Comparisons of the relative abundances of differentially abundant families and genera with a prevalence >10% and a maximum proportion >0.2% among the M0, M2 and M4 groups (p<0.05). (E) Comparisons of the relative abundances of additional differentially abundant families and genera with a prevalence >10% among the M0, M2 and M4 groups (p<0.05). (F) Comparisons of the relative abundance of additional differentially abundant genera (relatively discrete) with a prevalence >10% among the M0, M2 and M4 groups (p<0.05). (G) Comparisons of the relative abundance of differentially abundant genera with a prevalence >10% and a maximum proportion >0.2% among the P0, P2 and P4 groups (p<0.05). (H) Comparisons of the relative abundances of additional differentially abundant phyla, families and genera with a prevalence >10% among the P0, P2 and P4 groups (p<0.05). (I) Comparisons of the relative abundances of additional differentially abundant families (relatively discrete) with a prevalence >10% among the P0, P2 and P4 groups (p<0.05). (J) Comparisons of the relative abundances of additional differentially abundant genera (relatively discrete) with a prevalence >10% among the P0, P2 and P4 groups (p<0.05). ^*^p indicates a p value <0.05 among the three groups, p^*^ indicates a p value <0.05 between each pair of groups. *, p value <0.05; **, p value <0.01; and ***, p value <0.001.

**Fig. S5 Correlation between differential microbiota and basal clinical indicators.**


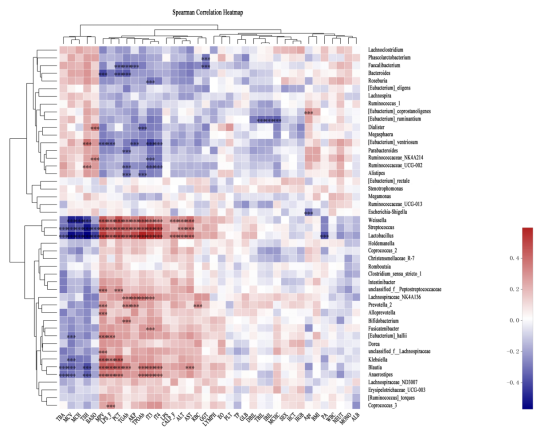


Color intensity represents the magnitude of correlation. Red, positive correlations; blue, negative correlations. * p value <0.05; ** p value <0.01; *** p value <0.001.

**Fig. S6 Basic index and gut microbiota changed after ATDs administration in SD rats**


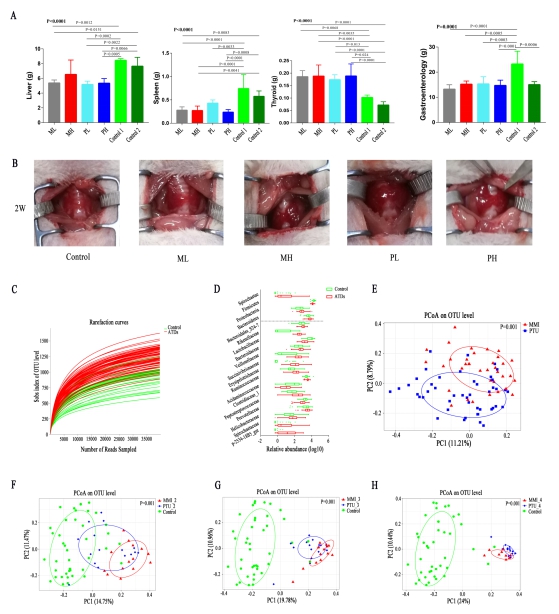


1. Final organ (liver, spleen, thyroid and gastrointestinal) weights in rats treated with ATDs. The liver and spleen weights were reduced in the ATDs group, while the thyroid weight was increased, and the gastrointestinal tissue weight was significantly correlated with body weight. (B) Thyroid gland appearance in living SD rats after 2 weeks of ATDs administration. The thyroid volume was enlarged in the ATDs group, and it was even more enlarged in the high-dose group. (C) Rarefaction curves for each rat sample were nearly smooth, with a sufficient amount of sequencing data and few new undetected genes. (D) Venn diagram demonstrating the number of shared and specific OTUs among the ATD and control groups. (E) PCoA based on the Bray-Curtis distance showing different clusters among the MMI and PTU groups (p=0.001). (F-H) PCoA based on the Bray-Curtis distance showing different clusters among the MMI and PTU subgroups at specific time points.

**Fig. S7 Change of gut microbiota structure among different drug and different dose subgroups.**


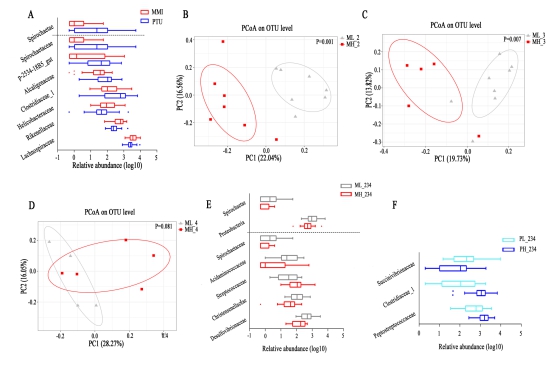


(A) Comparisons of the relative abundances of differentially abundant phyla and families among the MMI and PTU groups by Mann-Whitney U-tests (p<0.05). (B-D) PCoA based on the Bray-Curtis distance among the low-dose MMI and high-dose MMI subgroups at specific time points. (E) Comparisons of the relative abundances of differentially abundant phyla and families among the ML_234 and MH_234 groups by Mann-Whitney U-tests (p<0.05). (F) Comparisons of the relative abundances of differentially abundant phyla and families among the PL_234 and PH_234 groups by Mann-Whitney U-tests (p<0.05).

**Fig. S8 Longitudinal differentially abundant phyla and families among different subgroups.**


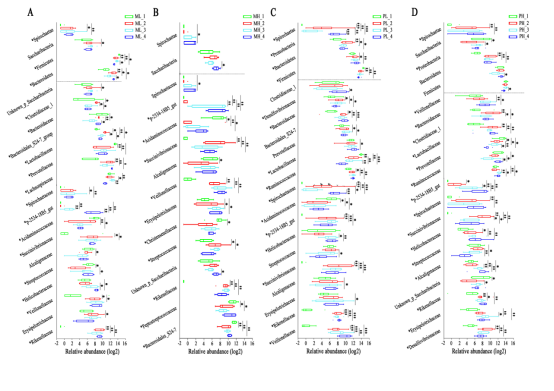


(A-D) Comparisons of the relative abundances of longitudinal differentially abundant phyla and families among the ML, MH, PL and PH groups (p<0.05). ^*^p indicates a p value <0.05 among the three groups, p^*^ indicates a p value <0.05 between each pair of groups. *, p value <0.05; **, p value <0.01; and ***, p value <0.001.

**Fig. S9 Longitudinal differentially abundant genera among different subgroups.**


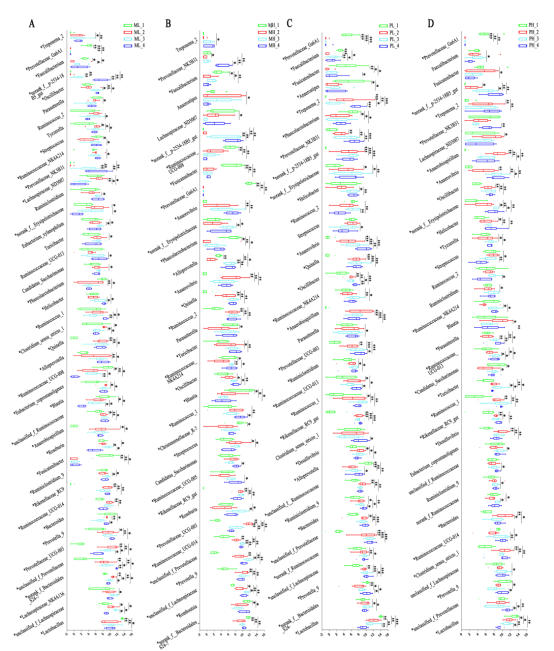
(A-D) Comparisons of the relative abundances of longitudinal differentially abundant genera among the ML, MH, PL and PH groups (p<0.05). ^*^p indicates a p value <0.05 among the three groups, p^*^ indicates a p value <0.05 between each pair of groups. *, p value <0.05; **, p value <0.01; and ***, p value <0.001.

**Fig. S10 Correlation between differential microbiota and basal clinical indicators.**


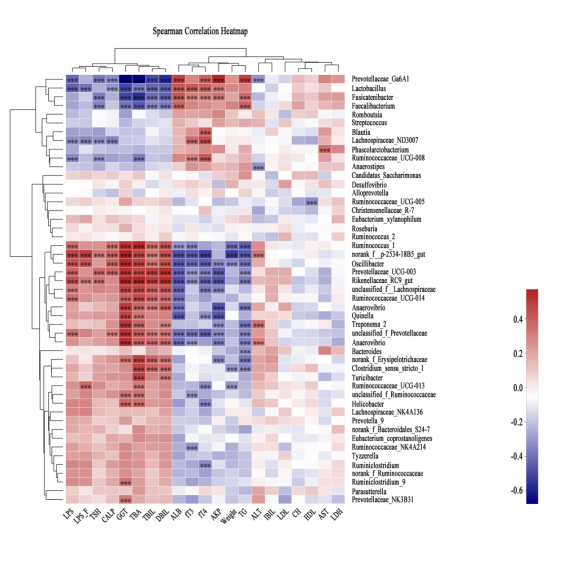


Color intensity represents the magnitude of correlation. Red, positive correlations; blue, negative correlations. * p value <0.05; ** p value <0.01; *** p value <0.001.
